# Shedding Light on the Role of Neurotransmitters in the Microenvironment of Pancreatic Cancer

**DOI:** 10.3389/fcell.2021.688953

**Published:** 2021-07-30

**Authors:** Yiyi Liang, Huimin Li, Yu Gan, Hong Tu

**Affiliations:** State Key Laboratory of Oncogenes and Related Genes, Shanghai Cancer Institute, Renji Hospital, Shanghai Jiao Tong University School of Medicine, Shanghai, China

**Keywords:** pancreatic cancer, tumor microenvironment (TME), neurotransmitter, immunotherapy, immune cells

## Abstract

Pancreatic cancer (PC) is a highly lethal malignancy with a 5-year survival rate of less than 8%. The fate of PC is determined not only by the malignant behavior of the cancer cells, but also by the surrounding tumor microenvironment (TME), consisting of various cellular (cancer cells, immune cells, stromal cells, endothelial cells, and neurons) and non-cellular (cytokines, neurotransmitters, and extracellular matrix) components. The pancreatic TME has the unique characteristic of exhibiting increased neural density and altered microenvironmental concentration of neurotransmitters. The neurotransmitters, produced by both neuron and non-neuronal cells, can directly regulate the biological behavior of PC cells via binding to their corresponding receptors on tumor cells and activating the intracellular downstream signals. On the other hand, the neurotransmitters can also communicate with other cellular components such as the immune cells in the TME to promote cancer growth. In this review, we will summarize the pleiotropic effects of neurotransmitters on the initiation and progression of PC, and particularly discuss the emerging mechanisms of how neurotransmitters influence the innate and adaptive immune responses in the TME in an autocrine or paracrine manner. A better understanding of the interplay between neurotransmitters and the immune cells in the TME might facilitate the development of new effective therapies for PC.

## Introduction

Although the accumulation of genetic and epigenetic defects is believed to drive carcinogenesis, the progression of cancer is indeed highly dependent on the interactions between cancerous and non-cancerous cells in the tumor microenvironment (TME). Immune cells make a large contribution to the non-malignant cellular components in the TME (Ho et al., 2020). Tumor-infiltrated immune cells bring out an important and complicated regulatory function in cancer progression. The TME also includes multiple secreted non-cellular components, such as cytokines, neurotransmitters and extracellular matrix (Dey et al., 2020; Winkler et al., 2020). Among them, neurotransmitters are recently emerging as a novel non-cellular portion of the TME that have been appreciated in cancer progression, especially in pancreatic cancer (PC) (Tan et al., 2021).

Pancreatic cancer is a devastating malignant disease with a very dismal prognosis (Ligorio et al., 2019). PC has a unique TME characterized by a markedly increased neural density. Neural remodeling and perineural invasion (PNI), the term describing the neoplastic invasion of tumor cells into nerves, are two common adverse histological characteristics of PC. As a group of chemical substances released by neurons, neurotransmitters have been documented to play a vital role in PC (Renz et al., 2018b). Altered concentration of several neurotransmitters is usually observed in the TME of PC and is associated with increased cancer aggressiveness and worsened overall prognosis (Biffi et al., 2019; Jurcak and Zheng, 2019; Hosein et al., 2020). PC cells showed chemotaxis toward neurotransmitters. Neurotransmitters can directly regulate the biological behavior of PC cells via binding to their corresponding receptors on tumor cells and activating the intracellular downstream signals (Entschladen et al., 2008). Moreover, recent studies have revealed that neurotransmitters do not only act on cancer cells, but also communicate with the immune cells in the TME (Binnewies et al., 2018), which suggests an indirect role of neurotransmitters in regulating the fate of PC by the crosstalk between neurotransmitters and the immune microenvironment in PC.

In this review, we will summarize the present progresses on the functions of neurotransmitters in the TME of pancreatic cancer. We will not only present the literatures that support direct effects of neurotransmitters on PC cells, but also discuss the interplay between neurotransmitters and the tumor immune microenvironment. Lastly, we will provide our perspectives on the potential therapeutic strategies the targeting neurotransmitter-immune cell crosstalk in PC.

## Non-Neurological Roles of Neurotransmitters in Cancers

### Classification, Origin and Operation of Neurotransmitters

Neurotransmitters are biochemical molecules that carry information between neurons or between neurons and effector cells (Orrego, 1979). Most neurotransmitters are typically water-soluble molecules with dissociating groups. Based on their chemical structure, the critical classification of neurotransmitters can be summarized as follows (Spitzer, 2015): biogenic amines, amino acids, peptides, and other categories. Biogenic amine neurotransmitters are composed of dopamine (DA), norepinephrine (NE), epinephrine (E), and serotonin (5-HT). Amino acid neurotransmitters contain gamma-aminobutyric acid (GABA), glycine, glutamate, histamine, and acetylcholine (Ach). Peptide neurotransmitters include substance P (SP), neuropeptide Y (NPY), calcitonin gene related peptide (CGRP) and many others. Also, neurotransmitters can be classified by their function (excitatory or inhibitory) or by their action (direct or neuromodulator). Excitatory neurotransmitters (such as NE) activate the postsynaptic neuron and facilitate interneuronal information transduction, while inhibitory neurotransmitters (such as GABA) inhibit the postsynaptic neuron and hinder information transduction. Some neurotransmitters can be both excitatory and inhibitory. Neuromodulators (such as 5-HT and DA) do not directly participate in interneuronal information transduction, but work together with excitatory or inhibitory neurotransmitters to modify the postsynaptic cell’s response (Boto and Tomchik, 2019). Differ from neuromodulators in the extent of actions, neurohormones (such as oxytocin and vasopressin) are secreted by neurosecretory cells into the blood steam and exert their effect on distant peripheral targets (Iovino et al., 2019).

Emerging evidence demonstrates that neurotransmitters can be released not only by the neurons from the central or peripheral nervous system, but also by non-neuronal cells (Table 1). For instance, Ach, the first established neurotransmitter, has been found to be synthesized in a variety of non-neuronal cells such as epithelial cells (from airway, digestive tract, urogenital tract, or epidermis), mesothelial cells (from pleura or pericardium), endothelial cells, fat cells, and fibroblasts (Reijmen et al., 2018; Elyada et al., 2019). The primary and secondary lymphatic organs of the immune system can be innervated by nerves. The local neurotransmitter thus can act as an immunomodulatory messenger to regulate the interaction between peripheral nerves and the lymphocytes (Zhang et al., 2014; Blanchart et al., 2017; Sung et al., 2017; Hujber et al., 2018). This peripheral innervation has been reported to participate in the development of immune cells (Allen et al., 2020). Meanwhile, the immune cells can also produce specific neurotransmitters such as 5-HT (Chen et al., 2015) to regulate the immune cells function and remodel the surrounding microenvironment through autocrine and paracrine approaches (Briggs et al., 2016).

**TABLE 1:** The origin of neurotransmitters.

Neurotransmitters	Non-neuronal secretion	References
Norepinephrine	Thymic cells	Roggero et al., 2011; McBurney-Lin et al., 2019
Serotonin	Enterochromaffin (EC) cells of the gastrointestinal tract, thymic epithelial cells, cancer cells, immune cells, platelet	Lifantseva et al., 2017; Spohn and Mawe, 2017
Dopamine	Gastrointestinal tract, thymic epithelial cells, immune cells	Lifantseva et al., 2016; Liu C. et al., 2021
GABA	Cancer cells, the endocrine cells of pancreatic islets	Soltani et al., 2011; Kim et al., 2015
Substance P	Enterochromaffin (EC) cells of the gastrointestinal tract, thymic epithelial cells	Heitz et al., 1976; Zaidi and Matthews, 2013
NPY	Cancer cells	Gotzsche and Woldbye, 2016
CGRP	Immune cells	Bracci-Laudiero et al., 2002; Messlinger, 2018
Acetylcholine	Epithelial cells, immune cells, cancer cells	Brenner and Sakmann, 1978; Kawashima and Fujii, 2004
Histamine	Mast cells	Haas et al., 2008; Misto et al., 2019
Glutamate	Endocrine cells of pancreatic islets	Cabrera et al., 2008

In the nervous system, neurotransmitters mediate interneuronal communication in synaptic transmission. Although whether nerve-to-non-neuronal cell synapses or synapse-like structures exist outside of the nervous system is not yet known, the nervous system can influence non-neuronal cells through changing circulating neurotransmitter levels (Monje et al., 2020). In the tumor microenvironment, neurotransmitters may be also secreted from non-neuronal cells and confer both paracrine and autocrine effects on cancer cells, as well as immune cells.

### The Role of Neurotransmitters in Cancer

Similar to the process of neovascularization and lymphangiogenesis, the formation of new nerve endings in the tumor is called neurogenesis. Neurogenesis is one of the determinants in tumorigenesis and cancer development (Boilly et al., 2017). Neurotransmitters serve as a link between intratumoral nerves and tumor cells in the TME. Tumor cells express various neurotransmitter receptors. Neurotransmitters released from nerve fibers in the TME can directly act on tumor cells by binding to their specific neurotransmitter receptors (Hanoun et al., 2015; Renz et al., 2018a,b). Meanwhile, tumor cells can also produce endogenous neurotransmitters in response to diverse stimuli from the microenvironment. For example, various types of tumor cells have been revealed to produce GABA. The elevated intratumoral level of GABA has been observed in PC as well as ovarian cancer and breast cancer (Zhang et al., 2014; Jiang et al., 2019).

Neurotransmitters can affect almost all aspects related to tumor development including cell proliferation, angiogenesis, and metastasis (Boilly et al., 2017). Peripheral 5-HT generates a mitotic effect on a variety of tumor and non-tumor cells such as fibroblasts, smooth muscle cells, osteoblasts, mesangial cells, and endothelial cells (Alpini et al., 2008; Moon et al., 2020). Many studies have shown a potential stimulatory effect of 5-HT on cancer cell proliferation, invasion, dissemination, and tumor angiogenesis (Herr et al., 2017). Abnormal glutamate signaling showed carcinogenic potential in glioma, melanoma, breast cancer, and prostate cancer (Ribeiro et al., 2017; Sung et al., 2017; Yu et al., 2017; Anastas and Shi, 2019). Substance P and SP/NK-1 system have also been involved in the development and progression of many cancers such as glioma, colon cancer, and lung cancer (Munoz et al., 2011; Covenas and Munoz, 2014). Elucidating their specific roles in tumor biology especially in the TME may open up new windows for the diagnosis and treatment of cancers.

## Neurotransmitters in the Tme of Pancreatic Cancer

### Nerves and Neurotransmitters Are Key Components of Pancreatic TME

The TME of pancreatic cancer is characterized by nutrient deficiency, connective tissue hyperplasia and high nerve distribution. Paracrine signals derived by cancer cell promote nerve axonogenesis or neurogenesis in the TME. The infiltrated nerve fibers can control cancer initiation, growth and metastasis. Nerve fibers in the pancreatic TME include axons originating from the sympathetic, parasympathetic, enteropancreatic or hepatic plexus, afferent nerve fibers and newly developed nerve fibers. Neuron as presynaptic cell can secrete neurotransmitters such as E/NE, which act on specific receptors to regulate tumor proliferation and metastasis. In PC cells, sensory (Saloman et al., 2016; Sinha et al., 2017) and sympathetic nerves activate the growth of PC cells through the liberation of substance P and E/NE. On the contrary, parasympathetic nerves inhibit PC cell growth via ACh, leading to the inhibition of PI3K/AKT and EGFR/ERK in cancer cell (Renz et al., 2018b). This opposite impact of sympathetic and parasympathetic nerves suggests that the development of PC is regulated through a balance of neural innervation.

Neural remodeling and PNI are important pathological characteristics of PC (Guo et al., 2013). Neural remodeling is manifested as the increased size and density of infiltrated nerves in the pancreas. PNI is considered as one of the main routes for PC recurrence and metastasis after surgical resection since it presents a structural conduit for cell migration. Recent studies have illustrated that multiple types of cells in the TME of PC contribute to neural remodeling and PNI (Li et al., 2014). New nerve fibers in the TME act as a rich source of neurotransmitters and neurotrophic factors (Zahalka and Frenette, 2020), which substantially affects the malignant potential of tumor cells and the disease progression. For example, the classical neurotransmitters NE and 5-HT have been found to be significantly increased in PC tissues (Zhao et al., 2014; Jiang et al., 2017), and the altered levels of different neurotransmitters have been demonstrated to be associated with PC recurrence, metastasis, and survival (Guo et al., 2013), suggesting that microenvironmental neurotransmitters function as an essential non-cellular component that contributes to PC progression (Schuller et al., 2012).

### Direct Effects of Neurotransmitters on Pancreatic Cancer

Neurotransmitters in the TME can be released by tumor-infiltrating fibers, cancer cells and non-cancerous cells such as immune cells and epithelial cells (Entschladen et al., 2008; Boilly et al., 2017). Neurotransmitters have numerous regulatory functions on PC cells, which we summarized in Table 2.

**TABLE 2:** Summary of the studies on the role of neurotransmitters in pancreatic cancer.

Neurotransmitters	Receptor	Effect on pancreatic cancer cells	PC cells or animal models	References
Norepinephrine	α, β adrenergic receptors	Norepinephrine promotes proliferation, migration, invasion and inhibits apoptosis of pancreatic cancer cell	Panc-1, MIAPaCa-2, BxPC-3, AsPc-1, HPAC, SW1990 cells	Huang et al., 2012; Guo et al., 2013; Qian et al., 2018
Serotonin	5-HT receptors	5-HT increased proliferation and prevented apoptosis of PDAC cell lines	Kras/Trp53/Pdx1-Cre (KPC) mice, BxPC-3, HPAC, Panc-1, and SW1990 cells	Jiang et al., 2017
Dopamine	D1-like receptors D2-like receptors	The antagonists of DRD2, pimozide and haloperidol, inhibited the proliferation and migration of pancreatic cancer cells	BxPC-3, Panc-1, MIAPaCa-2, Capan-1, CFPAC-1 cells	Jandaghi et al., 2016
GABA	GABA_*A*_, GABA_*B*_	GABA stimulates pancreatic cancer growth through overexpressing GABAA receptor pi subunit	KLM-1, SUIT-2, KP-1N, PK-1, PK-45P, PK59, MIA PaCa-2, Panc-1 cells	Takehara et al., 2007
Substance P	NK-1 receptor	SP induces pancreatic cancer cell proliferation and invasion via NK-1 receptor and the high expression of NK-1receptor was associated with poor prognosis in patients	CAPAN-1, ASPC-1, PA-TU 8902, BxPC-3, MIAPaCa-2 cells	Munoz and Covenas, 2014, 2015
NPY	Y1-4 receptors	Y2 is strongly overexpressed in pancreatic cancer and may modulate angiogenesis	LsL-Kras^*G12D*^, LsL-Trp53^*R172H*^, Pdx1-Cre (KPC) mice	Zhou, 1993; Waldmann et al., 2018
CGRP	Calcitonin-like receptor (CLR). Receptor activity-modifying protein 1 (RAMP1). Receptor component protein (RCP)	CGRP stimulates the growth of PU-PAN-1 tumor cells, and CGRP receptor antagonist CGRP8-37 inhibited this effect	PU-PAN-1 cells	
Acetylcholine	nAchR, mAchR	Administration of a muscarinic agonist suppresses pancreatic cancer tumorigenesis	LSL-Kras^+/G12D^; Pdx1-Cre (KC) and LSL-Kras^+/G12D^; LSL-Trp53^+/R172H^; Pdx1-Cre (KPC) mice, Panc-1 cells	Renz et al., 2018b
Histamine	Histamine1-4 receptors	When bound to H1HR, histamine induces proliferation and metastasis of PANC-1 cells. Activation of H2HR in PANC-1 cells tends to have the opposite effect of H1HR activation.	Panc-1 cell	Francis et al., 2013
Glutamate	AMPA receptors. Kainite receptors. NMDA receptors.	Glutamate increases pancreatic cancer cell invasion and migration	Su86.86 cells, BxPC-3 cells	Herner et al., 2011

#### Epinephrine and Norepinephrine

The classical neurotransmitters E and NE have been found to give promotion to PC progression through multiple mechanisms. E and NE are stress molecules produced by the sympathetic nervous system and linked to PC growth via β-adrenergic signaling in both *in vitro* and *in vivo* studies (Renz et al., 2018b). Specifically, E indirectly enhanced β-AR-dependent neurotrophin secretion, which in turn increased NE levels and promoted PC growth (Zhao et al., 2014). The activation of β-AR can promote tumor growth and angiogenesis via VEGF and metalloproteinase MMP2/MMP9 signaling pathways (Thaker et al., 2006). NE also tended to promote PC progression through β-AR/PKA/STAT3 signaling pathway (Coelho et al., 2017).

#### Serotonin

The neurotransmitter 5-HT, as well as its receptors, was found to be elevated in PC tissues (Jiang et al., 2017). Knockdown of 5-HT receptors inhibited the proliferation and invasion of human PC cells *in vitro* (Gurbuz et al., 2014). In contrast, the activation of 5-HT receptors enhanced glycolysis under metabolic stress, and thus promoted the growth of PC. Regarding to its molecular mechanism, 5-HT stimulation increased the Warburg effect through PI3K-Akt-mTOR signaling (Jiang et al., 2017). In addition, the increased levels of type 1 tryptophan hydroxylase (TPH1), which was a key enzyme for peripheral 5-HT synthesis, and the decreased level of MAOA, which is responsible for 5-HT degradation, in PC tissues were correlated with the poor survival of patients (Jiang et al., 2017). Of importance, the metaplasia of acinar-to-ductal metaplasia (ADM) is a key determinant in PC development (Liou et al., 2017). Serotonin uptake by acinar cells could promote the activation of the small GTPase Ras-related C3 botulinum toxin substrate 1 (Rac1), which is required for the transdifferentiation of acinar cells into ADM (Saponara et al., 2018).

#### Dopamine

The effect of DA on cancer cells is tumor type-specific. DA mainly reduced the proliferation and migration of endothelial cells in TME (Hoeppner et al., 2015). In PC, the dopamine receptor D2R is abnormally highly expressed and the antagonists of D2R (pimozide and haloperidol) were able to prevent the proliferation of PC cells, suggesting a PC-promoting effect of DA (Jandaghi et al., 2016).

#### Gamma-Aminobutyric Acid

Gamma-aminobutyric acid is a major inhibitory neurotransmitter in the central nervous system (CNS). Different GABA receptors play different roles in tumor growth. GABA was found to enhance prostate cancer cell proliferation through the GABA-A receptor pathway (Blanchart et al., 2017) and to inhibit cancer cell growth through the GABA-B receptor pathway in liver cancer (Wang et al., 2008; Hujber et al., 2018). GABRP, a subunit of the GABA-A receptor, was abnormally highly expressed in PC cells (Jiang et al., 2019). GABA treatment in GABRP-positive PC cells increased intracellular Ca^2+^ levels and activated the MAPK/Erk cascade, which led to a pro-tumor effect on PC (Takehara et al., 2007).

#### Neuropeptides

Neuropeptides, such as substance P, CGRP, and NPY, were also found to have a direct effect on PC cells. Substance P was a powerful regulator to PNI in PC during the early stage of primary tumor formation via the MMP1/PAR1/SP/NK-1R paracrine loop (Huang et al., 2018). Besides, substance P induced cancer cell proliferation and invasion as well as the expression of MMP-2 in PC cells, and sensory nerves in TME may help PC progression in part through up-regulation of its receptor (Sinha et al., 2017). Human PC cells possess distinct CGRP receptors. CGRP can stimulate the proliferation of human PC cells, suggesting a role of CGRP in the growth of PC cells (Zhou, 1993). What’s more, CGRP and substance P derived from pancreatic stellate cells mediated the PC pain via activation of sHH signaling pathway (Han et al., 2016), which provided a novel therapeutic option for PC pain. NPY could be detected in both human and murine pancreatic samples. Its receptor Y2 was significantly increased in PanIN lesions and PC samples both in murine and human. The enhanced Y2 receptor-mediated NPY signaling may modulate the angiogenesis of PC (Waldmann et al., 2018).

### Effects of Neurotransmitters on Non-malignant Cells in Pancreatic TME

In addition to affecting tumor cells, neurotransmitters can also improve angiogenesis, lymphangiogenesis, and inflammatory responses via exerting influence on endothelial cells and stromal cells in the TME. For instance, the activation of β-AR (β2 and β3) expressed on stromal cells promotes the survival of prostate cancer cells via TGF-β signaling (Magnon et al., 2013). Neovascularization is a vital process involved in tumor growth and metastasis. There is substantial evidence indicating that vascular endothelium infiltrated in the TME expresses various neurotransmitter receptors such as E and NE (Sarkar et al., 2013). NE could also stimulate endothelial cell metabolism and drive angiogenesis in tumors (Zahalka et al., 2017; Hondermarck and Jobling, 2018). DA was shown to mobilize endothelial progenitor cells from the bone marrow and participate in angiogenesis in the TME (Chakroborty et al., 2008). Neuropeptide Y released by tumor cells interacts with receptors on endothelial cells or immune cells, modulating tumor-related angiogenesis and local inflammatory responses (Medeiros and Jackson, 2013). Meanwhile, neurotransmitters can also regulate stromal cells in the microenvironment. Using a high-throughput drug screening system that focuses on the pancreatic stellate cells, Sagara et al. found that dopamine antagonist could inhibit the activation of pancreatic stellate cells and suppressed the invasion of pancreatic cancer cells by disrupting tumor-stromal interaction (Sagara et al., 2021). In addition, 5-HT was demonstrated to be essential for the survival and activation of hepatic stellate cells. Serotonin-activated stellate cells could promote carcinogenesis and contribute to sex disparity in hepatic cell carcinoma (Yang et al., 2017).

## Interplays Between Neurotransmitters and the Immune Cells in Pancreatic Tme

### Immune Cells in the TME of Pancreatic Cancer

Intratumoral immune heterogeneity is considered as a hallmark feature of the TME (Riggan et al., 2021). The tumor immune microenvironment has an immense influence on tumor initiation, progression and therapeutic response. Immune effector cells such as CD8^+^ cytotoxic T cells (CTL) and natural killer (NK) cells infiltrated in the TME keep the malignant cells under surveillance and form “barriers” to restrain cancer cell metastasis (Kurtulus et al., 2019). The secretion of multiple cytokines in pancreatic TME effect T helper (TH) cells, especially switching the balance of TH1/TH2, and contribute to the immunosuppressive microenvironment (Ho et al., 2020). Dendritic cells (DCs) are also considered as a significant component in adaptive anti-tumor immunity. DCs get involved in the proliferation of CTL in the TME (Puleo et al., 2018; Binnewies et al., 2019; Wculek et al., 2020) due to their role in tumor antigen recognition and presentation that stimulates T cell activation.

On the other hand, the TME can provide an immunosuppressive niche to negatively regulate immune effector cells and facilitate the malignant progression of cancer (Ho et al., 2020), and causes the resistance of PC against various treatments such as chemotherapy, targeted therapy, and immunotherapy (Balachandran et al., 2019). The major immunosuppressive cell types in the TME of pancreatic cancer are tumor-associated macrophages (TAMs), myeloid-derived suppressor cells (MDSCs), regulatory T cells (Treg), TH17, and tolerogenic DCs (Bronte and Tortora, 2016; Zhu et al., 2017; Ligorio et al., 2019; Zhang et al., 2020). These immunosuppressive cells promote tumor progression through a variety of mechanisms, including the direct mediation, inhibition of tumor-killing immune cells, induction of angiogenesis and lymphoangiogenesis (Ho et al., 2020), and promotion of metastasis. TAMs and MDSCs predominate in the TME and continuously communicate with PC cells to propagate disease progression (Lin et al., 2019). TAMs foster immune escape in the TME by suppressing TH1 cell and the antitumor responses of CTL. These contribute to matrix remodeling and facilitate tumor cell migration and invasion and promote tumor angiogenesis and growth (Qian and Pollard, 2010; Binnewies et al., 2018; Halbrook et al., 2019). MDSCs are known to exert immunosuppressive effects on T cells via secreting arginase, nitric oxide synthase, TGF-β, IL-10, and COX2 (Zhang et al., 2017). Tregs suppress tumor immunity in PC through a variety of pathways including the secretion of IL-10, TGF-β, and granzyme B, the activation of the TRAIL pathway and enhancement of T cells’ dysfunction (Zhang et al., 2020). TH17 cells are able to promote tumor cell growth by secreting IL-17, IL-23, and CCL20 (Wu H.H. et al., 2015), and inhibit the CD8^+^ T-mediated immune response by IL-17 and IL-22 (Yan and Richmond, 2020). Tumor-infiltrating lymphocytic B cells (TIL-B) resident in tertiary lymphoid structures were associated with better survival in PC patients, while TIL-B got involved in the initiation and progression of PC (Roghanian et al., 2016; Wouters and Nelson, 2018; Mirlekar et al., 2020).

As more TAMs were found in PC with PNI compared to that without PNI (Li et al., 2014; Alrawashdeh et al., 2019), the infiltration of immunosuppressive cells has been considered to relate to the PNI, which is the prominent characteristic of PC. Being the key molecular mediators of neuroimmune interactions, neurotransmitters might mediate the PNI-induced infiltration of immunosuppressive cells in PC. The PNI of pancreatic cancer could mediate β-AR signaling, and the released Ach enhanced tumor growth by establishing an immune-suppressive TME characterized by impaired CTL infiltration and a reduced TH1/TH2 ratio (Yang et al., 2020).

### Immune Cells as Non-neuronal Sources of Neurotransmitters

In recent years, promising studies have drawn the attention that immune cells in different activated states can synthesize or store neurotransmitters participate in neuroimmune regulatory circuits (Marino and Cosentino, 2013). Immune cells such as activated TH cells, Treg cells, and mature DC are able to synthesize or release serious classical neurotransmitters and their metabolites, including Ach, DA, and 5-HT. The synthesis of Ach was firstly observed in T cells (Ogawa et al., 2003). Compared with CD8^+^ T cells or B cells, CD4^+^ T cells contain more Ach (Kawashima and Fujii, 2003). TH cells could express tyrosine hydroxylase, the rate-limiting enzyme in the synthesis of DA, and store DA in the intracellular vesicles (Cosentino et al., 2000, 2007; Nakano et al., 2009). The inhibitory neurotransmitter such as 5-HT could be secreted by T cells, DCs, and macrophages (O’Connell et al., 2006; Wu et al., 2017).

Of interest, immune cells such as activated T cells have the capacity to synthesize 5-HT, and this potential is enhanced during their activation (O’Connell et al., 2006; Chen et al., 2015). CD8^+^ T cells were found to selectively express the highest level of type 1 tryptophan hydroxylase (TPH1), an enzyme that catalyzes the conversion of L-tryptophan (a direct precursor of 5-HT), indicating that they are capable of producing 5-HT. DCs and B cells can accumulate 5-HT through a regulated uptake mechanism from the microenvironment or activated T cells via serotonin transporters (SERTs) (Chen et al., 2015). Specifically, the expression of SERT on the surface of DC cells would increase as DCs matured or activated (Katoh et al., 2006), and also dynamically adjusted with the change level of 5-HT in the microenvironment (Arreola et al., 2015). Once DCs contact with T cells, their SERT expressions would significantly up-regulate (Chen et al., 2015). The stored 5-HT in DCs within LAMP-1^+^ vesicles were subsequently released via Ca^2+^-dependent exocytosis. Thus, DCs could sequester 5-HT, which are released from the microenvironment or directly from activated T cells, and transmit this 5-HT to naive T cells. This process suppressed cAMP production and thereby facilitated the activation and differentiation of naive T cells (O’Connell et al., 2006; Sacramento et al., 2018).

### The Regulatory Effect of Neurotransmitters on Immune Cells Infiltrated in the Pancreatic TME

**FIGURE 1:**
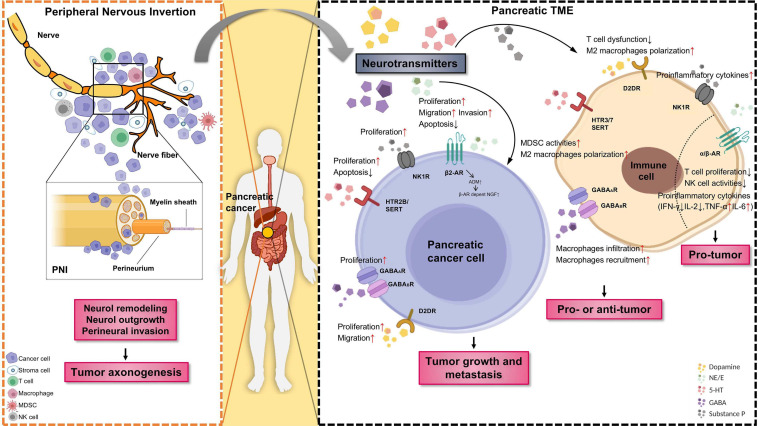
The role of neurotransmitters and the emergence of nerves in the pancreatic tumor microenvironment (TME). Tumor innervation is present as shown by the extension of nerve fibers into the solid tumor, while perineural invasion (PNI) is present as shown by the invasion of cancer cells into the perineurium of the nerve **(Left panel)**. The outgrowth of nerves in TME (axonogenesis) is partly driven by the secretion of neurotransmitters released by sympathetic nerves and other tissues or cells. In return, nerve fibers as branches of neuron infiltrate the TME, could regulate tumor growth and metastasis. Schematic representation of different neurotransmitters, their respective receptors, target cells as well as pro/antitumor activity of specific neurotransmitter/receptor axis **(Right panel)**. Neurotransmitters are an active component of the TME. Neurotransmitters released by neuro-endocrine-immune system and other tissues or cells can promote cancer cell proliferation, migration, and invasion through the stimulation of specific membrane receptors. Moreover, immune cells infiltrated in the TME likewise express diverse neurotransmitter receptors and react with neurotransmitter, are known to have a strong impact on tumor angiogenesis and inflammation. PNI, perineural invasion; TME, tumor microenvironment; MDSC, myeloid-derived suppressor cells. The figure was created with BioRender.com.

Neuro-immune interactions rely on soluble signaling molecules between cells, which including cytokines, chemokines, neurotransmitters, and neurotrophins. Neurotransmitters can regulate both the local and systemic immune responses against cancers. There is a comprehensive neuro-immune regulatory network existing in the TME, and the communication between neurotransmitters and immune cells influences the fate of cancer with either promoting or inhibiting the cancer growth and metastasis. The well-studied immune mediations caused by neurotransmitters are summarized in Table 3 and Figure 1. Understanding the regulatory effects of various neurotransmitters on cancer immunity can help to design new strategies for cancer therapy.

**TABLE 3:** Summary of the functions of neurotransmitters on immune cells.

Neurotransmitters	Immune cells	Effect	References
Epinephrine, norepinephrine	T-lymphocytes	Inhibit the activity of CD8^+^ T cells	Inbar et al., 2011; Dimitrov et al., 2019; Kim et al., 2019
	NK cells	Inhibit the activity of NK cells	
	Macrophages	Activation of β-AR signal could increase the infiltration of macrophages in the primary tumor parenchyma and induce the M2 polarization of macrophage, which subsequently promoted tumor metastasis	
Serotonin	T-lymphocytes	Releases IL-2, 16 and IFN-γ, T-cell proliferation	Uyttenhove et al., 2003; Yin et al., 2006; Mikulski et al., 2010; Inoue et al., 2011
	Dendritic cells	Increasing the Ca^2+^ concentration in immature cells	
		Activating 5-HT4 and 5-HT7 promotes the differentiation and maturation of DCs, up-regulates intracellular cAMP levels, and promotes the secretion of cytokines, such as IL-1β, IL-6, IL-8, and IL-10	
	Macrophages	Down-regulating chemokine CCR5 expression and up-regulating chemokine CCL2 and MIP1α expressions. Activating 5-HT2 increases the production and release of M2 cytokines. Inhibits release of TNF-α, inhibits NK cell suppression.	
	Neutrophils	Inhibits tumor cell phagocytosis and oxidative burst	
Dopamine	T-lymphocytes	T cells express functional dopamine receptors (DR) D1R-D5R, but their level and function are dynamic and context-sensitive; DA affects Th1/Th2/Th17 differentiation; D2 activation induces T cells to secrete IL-10;	Sarkar et al., 2006, 2013; Levite, 2016; Talhada et al., 2018
		D3 activation promotes T cells to secrete TNF-α, IFN-γ	
		D5 activation promotes T cells to secrete TNF-α and IL-10	
		Activation of D1 and D4 up-regulate the activity of cAMP and transcription factors STAT5 and GATA3, and promote the differentiation of T cells to TH2	
		DA inhibits the suppressive activity of Treg	
		DA activates resting effector T cells (Teffs) resulting in their proliferation and cytokine production. T cells produce DA (Tregs >>> Teffs), release DA, mainly after mitogen/antigen/CD3 ± CD28/PKC activation, uptake extracellular dopamine, and need DA. DA is important for antigen-specific interactions between T cells and dendritic cells.	
	Dendritic cells	Activating the D1 receptor on DC promotes ERK/JNK/NF-κB signaling, and inducing cytokine production	
	B-lymphocytes	Activating the D1 receptor on B cells increases the expression of its inducible ICOSL and CD40L	
GABA	T-lymphocytes Macrophages	Suppressing the immune effect by inhibiting the activation of NF-κB. Inhibiting the activation of macrophages by blocking calcium signals. Up-regulating the expression of the chemokines CXCL5 and CCL20.	Bergeret et al., 1998; Prud’homme et al., 2015
Substance P	T-lymphocytes	Affecting the activity and migration ability of CD8^+^ T cells	Mashaghi et al., 2016; Shahzad et al., 2018
	Dendritic cells	Regulating the movement of dendritic cells toward lymph nodes via modulating the expression of chemokine receptors and adhesion molecules.	
	Macrophages	Activating NF-κB signaling in macrophages increases the production of pro-inflammatory cytokines, and therefore amplified the inflammatory response mediated by Th1 or Th17	
	Peripheral blood monocytes	Producing pro-inflammatory cytokines, such as IL-1, IL-6, IL-12 and TNF-α	
	Neutrophils	Participating in migration of neutrophils	
NPY	T-lymphocytes	Promoting the polarization of Th2 polarizing by upregulating the production of IL-6 and IL-10	Wheway et al., 2007a,b
	Dendritic cells	Inducing migration of immature DCs through the engagement of NPY Y1 receptor and the activation of ERK and p38, and exerting proinflammatory effects through the recruitment of immature DCs	
CGRP	Dendritic cells	Inhibiting the capacity of dendritic cells to produce inflammatory cytokines and to present antigens to T cells	Fox et al., 1997; Holzmann, 2013
	Macrophages	Inhibition macrophages and upregulation of IL-10, IL-10-independent induction of the inducible cAMP early repressor (ICER) and inhibition of NF-κB activity	

#### Epinephrine and Norepinephrine

E and NE are mainly secreted by the adrenal medulla and sympathetic nerves, respectively. Local sympathetic innervation provides the bulk of the catecholamine content within the tumor (Zahalka and Frenette, 2020). Excessive activation of sympathetic nerves could damage the anti-tumor immune response, increase the invasion ability of tumor cells, and accelerate the occurrence and development of tumors (Nissen et al., 2018).

E and NE mainly exert immunosuppressive effects. The receptor β-ARs is present in most immune cells such as T cells (including Treg), B cells, macrophages, NK and DC cells (Sarkar et al., 2013). E and NE can inhibit the activity of CD8^+^ T and NK cells via inducing the apoptosis of these lymphocytes (Zhao et al., 2014). They are known to directly suppress the production of cytokines and T cell proliferation, and potently inhibit TCR-mediated integrin activation on human antigen-specific CD8^+^ T cells (Leone et al., 2015; Dimitrov et al., 2019). β-AR signal suppressed the production of IL-2 and IFN-γ and proliferation of CD4^+^ T cells depending on their stage of differentiation (Muthu et al., 2007). The combined application of endogenous E and prostaglandin could reduce the anti-tumor activity of NK cells, thereby promoting the progression of leukemia (Inbar et al., 2011; Meron et al., 2013). Besides, several studies have demonstrated that chronic adrenergic signaling suppresses NK cell activity in solid tumors (Melamed et al., 2005; Tarr et al., 2012; Graff et al., 2018). In addition, β-AR-mediated hormonal signaling could reduce the deformability of macrophages, resulting in the acceleration of tumor metastasis (Kim et al., 2019). NE and E contribute to the macrophage polarization, recruitment, and cytokine production like IL-6 and TNF-α (Izeboud et al., 1999; Chiarella et al., 2014). Activation of β-AR signal could increase the infiltration of macrophages in the primary tumor parenchyma and induce the M2 polarization of macrophages (Dimitrov et al., 2019), which subsequently promoted tumor metastasis and stimulated tumor cells to produce chemokines like M-CSF (Van Overmeire et al., 2016). The use of β-blocker propranolol reduced the immunosuppression function of MDSC in breast cancer and enhances the effect of other cancer therapies like anti-PD-1 treatment and irradiation (Mohammadpour et al., 2019).

In pancreatic TME, it was shown that NE and E could decrease the expressions of MHC-I molecules and the costimulatory ligand B7-1 in PC cells, and increase the expressions of immunosuppressive IDO, PD-1, and PD-L1 (Arreola et al., 2015). Although these changes only last for a short time, these phenotypic changes of cancer cells do not only prevent the antigen recognition by T cells, but also damage T cell function by depleting essential nutrients, such as tryptophan, and inducing the exhaustion of activated lymphocytes (Zhao et al., 2014). β-adrenergic activation directly inhibits the generation of CTL and blocks the recruitment of protective T cells in the tumor microenvironment (Nakai et al., 2014). In PC, stress-induced neural activation is related to increased primary tumor growth and tumor cell dissemination to the normal adjacent pancreas. These effects were associated with increased expression of invasion genes by tumor cells and pancreatic stromal cells in the microenvironment (Kim-Fuchs et al., 2014). Enriching the housing environment for mice could enhance the cytotoxic activity of NK cells and promote tumor-infiltrating NK cells via sympathetic nerve-dependent mechanisms. Application of the β-blocker largely abolished the effects of the enriched environment on NK cells and attenuated its anti-tumor function (Song et al., 2017).

Taken together, adrenergic signaling mainly exhibits pro-tumorigenic properties. This effect is generated in part through the enhanced immune evasion induced by E and NE. The inhibition of adrenergic signaling increases the antitumor immune response via its impact on multiple immune cells, supporting the potential value of adrenergic antagonists in cancer prevention and treatment.

#### Serotonin

In the brain, 5-HT is synthesized by neurons located in the raphe nucleus of the brainstem (Migliarini et al., 2013). Externally, less than 1% of free 5-HT exists in the blood, and the rest is stored in platelets, presynaptic neurons, and intestinal enterochromaffin cells (Kim et al., 2019). 5-HT is a multifunctional molecule that regulates immune function (Arreola et al., 2015). It is now known that there are seven different 5-HT receptor subtypes: 5-HT1 to 5-HT7. Except 5-HT3, which is a ligand-gated ion channel, all the other 5-HT receptors belong to the G protein coupled receptor family. Most immune cells express 5-HT receptors. 5-HT1 is mainly expressed in innate immune cells such as the mast cells (Kushnir-Sukhov et al., 2006), macrophages (Nakamura et al., 2008), DCs (Durk et al., 2005), and monocytes (Soga et al., 2007); 5HT-2 is expressed in eosinophils and macrophages (Mikulski et al., 2010). In the adaptive immune system, proliferated T cells mainly express 5-HT1B, 5-HT2A, and 5-HT7, and B cells mainly express 5-HT1A and 5-HT3 (Yin et al., 2006; Inoue et al., 2011).

5-HT is a neuromodulator with neurotransmitter and neuroendocrine functions in cancer (Balakrishna et al., 2021). Meanwhile, it also regulates a variety of immune processes, such as immune cell chemotaxis, activation, proliferation, and cytokine secretion (Herr et al., 2017). SERT blockers including the serotonin selective reuptake inhibitors (SSRI) which increase extracellular 5-HT concentration have immunoinhibitory effects (Gobin et al., 2014). In T cells, 5-HT induces T cell differentiation into Treg cells and promotes the shift of Th17 cells to Tregs. 5-HT acts on Th17 to induce the secretion of IFN-γ and IL-17 and elevates the release of IL-10 from Tregs, which indirectly promote the development of tumors (Sacramento et al., 2018). In DC cells, 5-HT elevates the Ca^2+^ concentration in immature cells, and contributes to the differentiation and maturation of DCs by activating 5-HT4 and 5-HT7. This activation could up-regulate cAMP levels in DCs (Carhart-Harris and Nutt, 2017) and the secretion of related cytokines such as IL-1β, IL-6, IL-8, and IL-10 (Idzko et al., 2004; Katoh et al., 2006). In macrophages, 5-HT regulates the polarization of macrophages through both activated and inhibitory signals. 5-HT2A activation increases the production of M2-type cytokines and migration while 5-HT1A activation enhances the capability of phagocytosis in macrophages (Mikulski et al., 2010; de las Casas-Engel et al., 2013). Activation of 5-HT1A on lung cancer cells could induce immune evasion via autophagy. After the activation, the ratio of TH1/TH2 cells decreased and the number of Tregs increased in TME, which suggesting a resistance to CTL attack (Liu et al., 2019).

In the pancreas, 5-HT is profoundly implicated in acute pancreatitis, pancreas regeneration after pancreatitis and PC. In the pancreatic TME, the SSRI fluoxetine reduced the stromal reaction that surrounds pancreatic lesions, evidenced by decreased fibrosis, inflammation and angiogenesis (Saponara et al., 2018). The intratumoral MAOA expression was also associated with T cell dysfunction and decreased patient survival in a broad range of cancer patients including PC (Wang et al., 2021).

In summary, in most cases, serotonin signaling influences immune cells and facilitates tumor development via suppression of anti-tumor immunity. Therefore, the use of anti-anxiety or anti-depressant drugs targeting serotoninergic system may have potential implications in cancer therapy.

#### Dopamine

Dopamine contributes to neuroimmune communication and acts on immune cells in an autocrine/paracrine manner through its receptors (Roy et al., 2000). Dopamine receptors are functionally classified into the D1-like subtypes consisting of D1R and D5R and D2-like subtypes (D2R, D3R, and D4R receptors), based on their ability to stimulate the formation or inhibition of cAMP (Kebabian, 1978).

Dopamine mainly acts on T cells and trigger DA receptor-dependent activation of ERK, LCK, FYN, and NF-κB pathways (Ghosh et al., 2003). In T cells, the activation of D2R and D5R can induce IL-10 (Besser et al., 2005; Cosentino et al., 2007), while D3R activation induces TNF-α and IFN-γ secretion (Ilani et al., 2004). Besides, the stimulation of D3R in naive CD8^+^ T cells also contributed to the regulation of chemotaxis and related cellular function like extravasation and adhesion (Huang et al., 2010; Figueroa et al., 2017). DA affects Th1/Th2/Th17 differentiation. Specifically, the activation of D1R and D4R enhanced the TH2 differentiation by up-regulating the activity of cAMP, STAT5, and GATA3. Besides, DA mediated the chemotactic migration of naive CD8^+^ T cells by inducing chemokines like CCL19, CCL21, and CXCL12 (Watanabe et al., 2006). It was reported that DA inhibits the proliferation of T cell and the secretion of IL-2, IL-6, and IFN-γ when exposed to a high level of DA (Bergquist et al., 1997; Saha et al., 2001). DA could activate D1-like receptors in Treg, which in turn indirectly activates effect T cell (Cosentino et al., 2007). Except for T cells, inhibition of the DR3-mediated signal in DC cells increased the cross-presentation of antigen to CD8^+^ T cells (Figueroa et al., 2017).

Dopamine functions as a regulatory component on immune cells in the TME (Yan et al., 2015; Petrilli, 2017). Increased level of plasma DA (40–80 pg/ml) was reported to evidently impair physiological proliferation and cytotoxicity of T cells in cancer patients (Saha et al., 2001). In PC, DA enhanced the chemotherapeutic efficacy of gemcitabine both *in vitro* and in immunocompetent murine models, and changed TME by suppressing the M2 characters of TAMs. Specifically, the activation of D4R in macrophages by DA reduced the production of cAMP, and then inhibited the activation of PKA/p38 signal pathway, which suppressed the transcription of tumor-promoting cytokines of TAMs such as IL-1β and TNF-α (Liu Q. et al., 2021). What’s more, DA was found to hinder the function of tumor-induced monocytic MDSCs on the proliferation and IFN-γ production of T cells in lung and melanoma cancer (Hoeppner et al., 2015; Wu J. et al., 2015). DA attenuated NO production by MDSCs directly, mediated by decreased iNOS expression and the downregulation of ERK and JNK signaling pathways. DA-induced activation of resting Teffs and suppression of Tregs seem beneficial for the immunotherapy of cancer. As for the receptors, D2R was identified as an upregulated protein in PC, and D2R antagonists (pimozide and haloperidol) reduced PC growth and particularly metastasis (Jandaghi et al., 2016). The anti-tumor efficacy of ONC201 and ONC212, two small molecule antagonists of D2R, were observed *in vivo* either administrated as a single agent or in combination with 5-fluorouracil, irinotecan, and oxaliplatin. When treated with ONC201, a broad induction of immune cytokines and effector molecules was observed among PC patients with longer progression-free survival (Lev et al., 2017; Stein et al., 2019). Besides, ONC201 has been reported to induce the proliferation of NK cells and activation via TRAIL and granzyme B in preclinical studies (Stein et al., 2019).

Together, the current findings suggest an important immunomodulatory effect of DA in cancer microenvironment. DA may confer immunopromoting or immunosuppressive effect dependent on the types of immune cells and the specific DA receptor they express.

#### Gamma-Aminobutyric Acid

Gamma-aminobutyric acid is the main inhibitory neurotransmitter in the central nervous system. In the periphery, GABA is produced by pancreatic β cells, T cells, and macrophages. These cells also express other components of the GABAergic system, including receptors, transporters, and metabolic enzymes (Wu et al., 2017). The GABAergic signaling system affects various functional characteristics of immune cells, such as antigen-induced T cell proliferation (Bjurstom et al., 2008) and LPS-induced cytokine release and effector T cell activity (Lang et al., 2003).

Both *in vivo* and *in vitro* studies have proved that GABA suppresses the immune effect by inhibiting the activation of NF-*κ*B and reducing the production of inflammatory cytokines (Bhat et al., 2010). As a negative regulator in the TME, GABA inhibits the activation of macrophages and T cells by blocking calcium signals and inhibiting NF-*κ*B pathway (Prud’homme et al., 2015). In addition, GABA was shown to regulate the expression of GABA-A receptor subunits on immune cells (Bergeret et al., 1998; Feske et al., 2012). In the pancreatic TME, GABRP expression was remarkably increased in PC tissues among other neurotransmitters’ receptors. GABRP expression correlated with macrophages density closely, and the deletion of macrophages largely abrogated the oncogenic functions of GABRP in PC. The GABRP on cancer cells promoted macrophages recruitment by inducing CXCL5 and CCL20 expression. Specifically, GABRP might act as a chaperone protein and regulate the activity of KCNN4 channel to induce Ca^2+^ signaling in PC cells. The GABRP-KCNN4 complex led to the activation of NF-κB, which further facilitated CXCL5 and CCL20 transcription to induce macrophage infiltration in PC (Jiang et al., 2019).

In general, GABA exerts immunosuppressive effects on diverse immune cells and blockade of GABA signaling in the microenvironment may improve the anti-tumor immune response against cancer cells.

#### Neuropeptides

Most studies related to the immunomodulatory effect of neuropeptides focused on the substance P. In the central system, substance P is released from the brain regions and regulates emotions and specific sensory nerve endings (Cunin et al., 2011; Munoz and Covenas, 2015). In the periphery, substance P is mainly secreted by immune cells such as macrophages and DC cells (Janelsins et al., 2013). Substance P exerts its biological activity through G protein-coupled neurokinin receptors (NKRs), namely NK-1R, NK-2R, and NK-3R (Suvas, 2017). NK-1R mainly exists in the immune system and mediates the effect of substance P on immune cells (Shahzad et al., 2018). Substance P acts on NK-1R to induce a local inflammatory environment in a concentration-dependent manner. Specifically, substance P mediates the migration, proliferation, and activation of immune cells. Substance P activated NF-κB signaling in macrophages, increased the production of pro-inflammatory cytokines such as CCL2, CXCL2, and IL-8 (Levite, 2008). Therefore substance P amplified the inflammatory response mediated by TH1 or TH17 (Cunin et al., 2011; Mashaghi et al., 2016). Recent studies illustrated that administration of substance P during the primary immune response amplifies the secondary immune response by activating CD8^+^ T cells (Ikeda et al., 2007). Substance P increased the migration of immune cells, including T cells and neutrophils, through a β-arrestin-dependent mechanism (Nichols et al., 2012). Substance P also stimulated human PBMC to produce pro-inflammatory cytokines including IL-1, IL-6, IL-12, and TNF-α (Cunin et al., 2011). As for innate immune cells, substance P activated NK cells and neutrophils by up-regulating their production of cytotoxic-associated molecules, such as perforin and granzyme (Fu et al., 2011; Mashaghi et al., 2016). However, the up-regulation of the NK-1R can be seen in both chronic pancreatitis and PC and enhanced NK-1R expressions were related to advanced tumor stage and a poorer prognosis (Li et al., 2013). In addition, CD10^+^ fibroblasts can inhibit squamous cancer cells invasion ability by diminishing substance P (Xie et al., 2010).

The peripheral sensory nerves, which mediate pain reflexes, may influence immune responses through the release of neuropeptides CGRP (Holzmann, 2013). CGRP directly acts on macrophages and dendritic cells and inhibits the capacity of these cells to produce inflammatory cytokines and to present antigens to T cells. The molecular mechanisms, by which CGRP acts on innate immune cells, include the upregulation of IL-10, IL-10-independent induction of the inducible cAMP early repressor (ICER) and inhibition of NF-κB activity (Fox et al., 1997). In addition, Liu et al. demonstrated that increased CGRP and neuronal p75 immune-reactivities in tumor-bearing mice, promoting chronic pain in bone metastasis (Liu et al., 2020).

Neuropeptide Y was found to induce a dose-dependent migration of immature DCs through the engagement of NPY Y1 receptor and the activation of ERK and p38. Meanwhile, NPY promoted the polarization of TH2 polarizing by upregulating the production of IL-6 and IL-10 (Wheway et al., 2007a,b). Thus, NPY may exert proinflammatory effects through the recruitment of immature DCs, but it may exert anti-inflammatory effects by promoting a TH2 polarization. In prostate cancer, depression-induced NPY secretion might promote the tumor infiltration of myeloid cells and therefore contribute to cancer progression (Cheng et al., 2019).

Taken together, the neuropeptides CGRP and NPY were suggested to impair the anti-tumor immunity and their inhibition may be a potential strategy for cancer treatment. On the contrary, substance P may facilitate the anti-tumor immune response. However, enhancing the substance P signaling in cancer cells could promote the progression of pancreatic cancer. The distinction between signaling mechanisms of substance P in immune cells and cancer cells warrants further studies.

## Neurotransmitter-Targeted Drugs as Combinatorial Strategies for Cancer Immunotherapy

The important role of neurotransmitters in cancer progression suggests that the drugs targeting neurotransmitter signaling may act as promising candidates for cancer treatment (Cole et al., 2015). In fact, the clinical trials of various neurotransmitter receptor antagonists or agonists are already ongoing (Table 4). Among them, β-blockers, which are the antagonists targeting adrenergic β receptors, are mostly studied (Botteri et al., 2013; Grytli et al., 2013a,b). Data from prostate cancer and breast cancer studies showed that patients using β-blockers, even former users, had significantly better survival outcomes than the non-users (Grytli et al., 2013b; De Giorgi et al., 2018). In melanoma, pan β-blockers provided more survival benefits than β1- or β2-selective blockers (Livingstone et al., 2013). In PC, long-term use of beta-blockers especially selective β1-blockers may be associated with decreased cancer risk (Saad et al., 2020). Besides, β-blocker drugs may lead to a significantly improved overall prognosis in PC patients, particularly among those with localized disease (Saad et al., 2020). These findings raise the possibility that neurotransmitters-related drugs protect from cancer initiation.

**TABLE 4:** Clinical trials related to neurotransmitters in cancer.

Related neurotransmitter	Cancer type and stage	Purpose	Strategy or treatment	Enrolled patients	Therapeutic effects	References
Catecholamine (E/NE)	Head and neck. Esophagus. Stomach. Colon. Prostate cancer.	Investigate the association between propranolol and cancer	Patients with a usage of propranolol >6 months in NHIRD database	24,238	HR:0.58 (95%CI: 0.35–0.95) HR:0.35 (95%CI: 0.13–0.96) HR:0.54 (95%CI: 0.30–0.98) HR:0.52 (95%CI: 0.33–0.83) Reduce cancer risk	Chang et al., 2015
Catecholamine (E/NE)	Locally advanced and metastatic melanoma	Investigate the efficacy of combination with propranolol and pembrolizumab	Propranolol twice a day with pembrolizumab 200 mg every 3 weeks	9	In progress (ORR = 78%) Responders show increased IFN-γ and decreased IL6 level	Gandhi et al., 2021
Serotonin	Breast cancer with obesity or/and overweight	Exam the effects of Mediterranean Diet and Naltrexone/Bupropion Treatment in Obese Breast Cancer Patients	Naltrexone/bupropion combination (NB)^+^ release (ER) combination tablets, Mediterranean Diet	72	The combination of the Mediterranean diet with naltrexone/bupropion treatment Without superior changes	NCT03581630 (Cho et al., 2020)
Dopamine	Pituitary adenoma; Non-functioning pituitary adenoma	Investigate the efficacy of cabergoline in NFPA individuals with remaining tumor after primary neurosurgery	Cabergoline (a DRD2 antagonist and antiparkinson drug)	140	Residual tumor shrinkage: 10.5% vs. 28.8%, stabilization = 66.1% vs. 73.7%, enlargement = 5.1% vs. 15.8% (the control group) PFS: 23.2M vs. 20.8M (the control group)	NCT03271918 (Batista et al., 2019)
Dopamine	Recurrent and stage IV breast cancer	Investigate the effectiveness of cabergoline in treating metastatic breast cancer disease in those who test positive for the prolactin receptor	Cabergoline (a DRD2 antagonist and antiparkinson drug)	20	CBR = 33% (6/18), mPFS = 1.8M, mOS = 10.4M	NCT01730729 (Costa et al., 2017)
Dopamine	Advanced solid cancer refractory to the standard treatment	Evaluate the safety and pharmacokinetics of weekly ONC201	ONC201 (a DRD2 antagonist)	20	Enhance immunostimulatory activity	NCT02250781 (Stein et al., 2019)

Recently, immunotherapy is emerging as the most promising treatment option for cancers. However, immunosuppressive mechanisms within the TME largely limit its therapeutic efficacy. As aforementioned, neurotransmitters not only have direct effects on tumor cells, but also contribute to the immunosuppressive microenvironment by acting on immune cells in the TME. Therefore, neurotransmitter-targeted drugs have attracted increasing attentions as a combinatorial approach for cancer immunotherapy. In murine tumor models, reducing β-AR signaling was shown to facilitate the conversion of TME to an immunologically active microenvironment, and β-blockers application significantly increased the efficacy of anti-PD-1 checkpoint blockade (De Giorgi et al., 2018). In addition, blocking β-AR signaling also improved the potency of TCR-γ T-cell therapeutics in hematologic malignancies (Baker et al., 2019) and enhanced the antitumor efficacy of STxBE7-based cancer vaccine in a breast cancer model (Daher et al., 2019). Small-molecule MAO inhibitors (MAOIs), used for depression and other neurological disorders, was found to significantly suppress tumor growth and generated synergistic tumor suppression effects when combined with anti-PD-1 treatment. Specifically, MAO-A restrains antitumor T cell immunity through controlling intratumoral T cell autocrine serotonin signaling. Apart from β-blockers and MAOIs (Wang et al., 2021), the results from preclinical studies provide a rationale for testing this combinatorial strategy in cancer patients.

The first prospective study on the combination of immunotherapy and a neurotransmitter receptor antagonist drug was conducted in melanoma patients (De Giorgi et al., 2018; Gandhi et al., 2021). It was shown that the patients taking propranolol, an approved non-selective β-blocker, not only had an 80% risk reduction for recurrence but also tended to be more sensitive to anti-PD-1 treatment (De Giorgi et al., 2018). The enhanced therapeutic efficacy of immune checkpoint inhibitors by β-blocker was also observed in lung cancer patients. The use of β-blockers was significantly associated with improved progression-free survival among non-small-cell lung cancer patients treated with SP-142, a PD-L1 inhibitor (Oh et al., 2021).

Pancreatic cancer is among the most immune-resistant tumor types. Given the potential role of neurotransmitters in the immunosuppressive microenvironment of PC, drugs targeting neurotransmitter signaling may help to limit immune suppression and overcome immunotherapy resistance in PC. The combination of neurotransmitter signaling-targeted drugs and immunotherapy is believed to provide new hope for PC patients (Balachandran et al., 2019).

## Conclusion

Pancreatic cancer has the unique characteristic of increased neural density. As the messenger molecules, neurotransmitters have emerged as components with significant importance in the TME of pancreatic cancer. Neurotransmitters can directly bind to the cancer cells, generating either promoting or inhibition effects on cancer growth. Meanwhile, the expressions of neurotransmitters and their corresponding receptors in the tumor-infiltrating immune cells imply a complicated relationship between the neurotransmitters and the immune cells in the TME. Further investigation on how neurotransmitters crosstalk with immune cells at the TME level will be of great interest, for it will facilitate our understanding of the mechanism behind the suppressive immune microenvironment of cancer. The tumor immune microenvironment contributes a lot to the initiation and development of cancers, as well as the response to cancer treatments, notably the immunotherapy. Nowadays, immunotherapy is increasingly involved in cancer therapeutic regimes. Breaking immune tolerance using specific receptor antagonists of neurotransmitters is possibly a promising strategy for combinatorial therapy with immune checkpoint inhibitors in PC. It is also plausible that modulating the expression of neurotransmitter receptors on the surface of CAR-T cells may promote the efficacy of CAR-T therapy. Further research into the precise mechanism of how immune cells regulated by different types of neurotransmitters in the TME might open up new avenues toward adjunct therapy against PC.

## Author Contributions

YL wrote the manuscript. HL searched the literatures, made the tables, and polished the language. HT and YG conceived the idea and revised the manuscript. All authors read and approved the final manuscript.

## Conflict of Interest

The authors declare that the research was conducted in the absence of any commercial or financial relationships that could be construed as a potential conflict of interest.

## Publisher’s Note

All claims expressed in this article are solely those of the authors and do not necessarily represent those of their affiliated organizations, or those of the publisher, the editors and the reviewers. Any product that may be evaluated in this article, or claim that may be made by its manufacturer, is not guaranteed or endorsed by the publisher.

## References

[B1] Allen B. M. Hiam K. J. Burnett C. E. Venida A. DeBarge R. Tenvooren I. (2020). Systemic dysfunction and plasticity of the immune macroenvironment in cancer models. *Nat. Med.* 26 1125–1134. 10.1038/s41591-020-0892-6 32451499 PMC7384250

[B2] Alpini G. Invernizzi P. Gaudio E. Venter J. Kopriva S. Bernuzzi F. (2008). Serotonin metabolism is dysregulated in cholangiocarcinoma, which has implications for tumor growth. *Cancer Res.* 68 9184–9193. 10.1158/0008-5472.CAN-08-2133 19010890 PMC2593938

[B3] Alrawashdeh W. Jones R. Dumartin L. Radon T. P. Cutillas P. R. Feakins R. M. (2019). Perineural invasion in pancreatic cancer: proteomic analysis and in vitro modelling. *Mol. Oncol.* 13 1075–1091. 10.1002/1878-0261.12463 30690892 PMC6487729

[B4] Anastas J. N. Shi Y. (2019). Histone serotonylation: can the brain have “happy” chromatin? *Mol. Cell* 74 418–420. 10.1016/j.molcel.2019.04.017 31051139 PMC6662934

[B5] Arreola R. Becerril-Villanueva E. Cruz-Fuentes C. Velasco-Velazquez M. A. Garces-Alvarez M. E. Hurtado-Alvarado G. (2015). Immunomodulatory effects mediated by serotonin. *J. Immunol. Res.* 2015:354957. 10.1155/2015/354957 25961058 PMC4417587

[B6] Baker F. L. Bigley A. B. Agha N. H. Pedlar C. R. O’Connor D. P. Bond R. A. (2019). Systemic beta-adrenergic receptor activation augments the ex vivo expansion and anti-tumor activity of Vgamma9Vdelta2 T-Cells. *Front. Immunol.* 10:3082. 10.3389/fimmu.2019.03082 32038628 PMC6993603

[B7] Balachandran V. P. Beatty G. L. Dougan S. K. (2019). Broadening the impact of immunotherapy to pancreatic cancer: challenges and opportunities. *Gastroenterology* 156 2056–2072. 10.1053/j.gastro.2018.12.038 30660727 PMC6486864

[B8] Balakrishna P. George S. Hatoum H. Mukherjee S. (2021). Serotonin pathway in cancer. *Int. J. Mol. Sci.* 22:1268. 10.3390/ijms22031268 33525332 PMC7865972

[B9] Batista R. L. Musolino N. R. C. Cescato V. A. S. da Silva G. O. Medeiros R. S. S. Herkenhoff C. G. B. (2019). Cabergoline in the management of residual nonfunctioning pituitary adenoma: a single-center, open-label, 2-year randomized clinical trial. *Am. J. Clin. Oncol.* 42 221–227. 10.1097/COC.0000000000000505 30540568

[B10] Bergeret M. Khrestchatisky M. Tremblay E. Bernard A. Gregoire A. Chany C. (1998). GABA modulates cytotoxicity of immunocompetent cells expressing GABAA receptor subunits. *Biomed. Pharmacother.* 52 214–219. 10.1016/S0753-3322(98)80019-X 9755818

[B11] Bergquist J. Josefsson E. Tarkowski A. Ekman R. Ewing A. (1997). Measurements of catecholamine-mediated apoptosis of immunocompetent cells by capillary electrophoresis. *Electrophoresis* 18 1760–1766. 10.1002/elps.1150181009 9372267

[B12] Besser M. J. Ganor Y. Levite M. (2005). Dopamine by itself activates either D2, D3 or D1/D5 dopaminergic receptors in normal human T-cells and triggers the selective secretion of either IL-10, TNFalpha or both. *J. Neuroimmunol.* 169 161–171. 10.1016/j.jneuroim.2005.07.013 16150496

[B13] Bhat R. Axtell R. Mitra A. Miranda M. Lock C. Tsien R. W. (2010). Inhibitory role for GABA in autoimmune inflammation. *Proc. Natl. Acad. Sci. U.S.A.* 107 2580–2585. 10.1073/pnas.0915139107 20133656 PMC2823917

[B14] Biffi G. Oni T. E. Spielman B. Hao Y. Elyada E. Park Y. (2019). IL1-induced JAK/STAT signaling is antagonized by TGFbeta to shape CAF heterogeneity in pancreatic ductal adenocarcinoma. *Cancer Discov.* 9 282–301. 10.1158/2159-8290.CD-18-0710 30366930 PMC6368881

[B15] Binnewies M. Mujal A. M. Pollack J. L. Combes A. J. Hardison E. A. Barry K. C. (2019). Unleashing type-2 dendritic cells to drive protective antitumor CD4(+) T cell immunity. *Cell* 177 556–571.e16. 10.1016/j.cell.2019.02.005 30955881 PMC6954108

[B16] Binnewies M. Roberts E. W. Kersten K. Chan V. Fearon D. F. Merad M. (2018). Understanding the tumor immune microenvironment (TIME) for effective therapy. *Nat. Med.* 24 541–550. 10.1038/s41591-018-0014-x 29686425 PMC5998822

[B17] Bjurstom H. Wang J. Ericsson I. Bengtsson M. Liu Y. Kumar-Mendu S. (2008). GABA, a natural immunomodulator of T lymphocytes. *J. Neuroimmunol.* 205 44–50. 10.1016/j.jneuroim.2008.08.017 18954912

[B18] Blanchart A. Fernando R. Haring M. Assaife-Lopes N. Romanov R. A. Andang M. (2017). Endogenous GABAA receptor activity suppresses glioma growth. *Oncogene* 36 777–786. 10.1038/onc.2016.245 27375015

[B19] Boilly B. Faulkner S. Jobling P. Hondermarck H. (2017). Nerve dependence: from regeneration to cancer. *Cancer Cell* 31 342–354. 10.1016/j.ccell.2017.02.005 28292437

[B20] Boto T. Tomchik S. M. (2019). The excitatory, the inhibitory, and the modulatory: mapping chemical neurotransmission in the brain. *Neuron* 101 763–765. 10.1016/j.neuron.2019.02.021 30844392

[B21] Botteri E. Munzone E. Rotmensz N. Cipolla C. De Giorgi V. Santillo B. (2013). Therapeutic effect of beta-blockers in triple-negative breast cancer postmenopausal women. *Breast Cancer Res. Treat.* 140 567–575. 10.1007/s10549-013-2654-3 23912960

[B22] Bracci-Laudiero L. Aloe L. Buanne P. Finn A. Stenfors C. Vigneti E. (2002). NGF modulates CGRP synthesis in human B-lymphocytes: a possible anti-inflammatory action of NGF? *J. Neuroimmunol.* 123 58–65. 10.1016/s0165-5728(01)00475-1 11880150

[B23] Brenner H. R. Sakmann B. (1978). Gating properties of acetycholine receptor in newly formed neuromuscular synapses. *Nature* 271 366–368. 10.1038/271366a0 304530

[B24] Briggs K. J. Koivunen P. Cao S. Backus K. M. Olenchock B. A. Patel H. (2016). Paracrine induction of HIF by glutamate in breast cancer: EglN1 senses cysteine. *Cell* 166 126–139. 10.1016/j.cell.2016.05.042 27368101 PMC4930557

[B25] Bronte V. Tortora G. (2016). Adipocytes and neutrophils give a helping hand to pancreatic cancers. *Cancer Discov.* 6 821–823. 10.1158/2159-8290.CD-16-0682 27485002

[B26] Cabrera O. Jacques-Silva M. C. Speier S. Yang S. N. Kohler M. Fachado A. (2008). Glutamate is a positive autocrine signal for glucagon release. *Cell Metab.* 7 545–554. 10.1016/j.cmet.2008.03.004 18522835 PMC4396785

[B27] Carhart-Harris R. L. Nutt D. J. (2017). Serotonin and brain function: a tale of two receptors. *J. Psychopharmacol.* 31 1091–1120. 10.1177/0269881117725915 28858536 PMC5606297

[B28] Chakroborty D. Chowdhury U. R. Sarkar C. Baral R. Dasgupta P. S. Basu S. (2008). Dopamine regulates endothelial progenitor cell mobilization from mouse bone marrow in tumor vascularization. *J. Clin. Invest.* 118 1380–1389. 10.1172/JCI33125 18340382 PMC2267013

[B29] Chang P. Y. Huang W. Y. Lin C. L. Huang T. C. Wu Y. Y. Chen J. H. (2015). Propranolol reduces cancer risk: a population-based cohort study. *Medicine (Baltimore)* 94:e1097. 10.1097/MD.0000000000001097 26166098 PMC4504645

[B30] Chen Y. Leon-Ponte M. Pingle S. C. O’Connell P. J. Ahern G. P. (2015). T lymphocytes possess the machinery for 5-HT synthesis, storage, degradation and release. *Acta Physiol. (Oxf.)* 213 860–867. 10.1111/apha.12470 25683571

[B31] Cheng Y. Tang X. Y. Li Y. X. Zhao D. D. Cao Q. H. Wu H. X. (2019). Depression-induced neuropeptide Y Secretion promotes prostate cancer growth by recruiting myeloid cells. *Clin. Cancer Res.* 25 2621–2632. 10.1158/1078-0432.CCR-18-2912 30504424

[B32] Chiarella S. E. Soberanes S. Urich D. Morales-Nebreda L. Nigdelioglu R. Green D. (2014). beta(2)-adrenergic agonists augment air pollution-induced IL-6 release and thrombosis. *J. Clin. Invest.* 124 2935–2946. 10.1172/JCI75157 24865431 PMC4071386

[B33] Cho A. R. Choi W. J. Kwon Y. J. Lee H. S. Ahn S. G. Lee J. W. (2020). Mediterranean diet and naltrexone/bupropion treatment for weight loss in overweight and obese breast cancer survivors and non-cancer participants: a pilot randomized controlled trial. *Diabetes Metab. Syndr. Obes.* 13 3325–3335. 10.2147/DMSO.S269237 33061494 PMC7532917

[B34] Coelho M. Soares-Silva C. Brandao D. Marino F. Cosentino M. Ribeiro L. (2017). beta-adrenergic modulation of cancer cell proliferation: available evidence and clinical perspectives. *J. Cancer Res. Clin. Oncol.* 143 275–291. 10.1007/s00432-016-2278-1 27709364 PMC11819197

[B35] Cole S. W. Nagaraja A. S. Lutgendorf S. K. Green P. A. Sood A. K. (2015). Sympathetic nervous system regulation of the tumour microenvironment. *Nat. Rev. Cancer* 15 563–572. 10.1038/nrc3978 26299593 PMC4828959

[B36] Cosentino M. Bombelli R. Ferrari M. Marino F. Rasini E. Maestroni G. J. (2000). HPLC-ED measurement of endogenous catecholamines in human immune cells and hematopoietic cell lines. *Life Sci.* 68 283–295. 10.1016/s0024-3205(00)00937-1 11191644

[B37] Cosentino M. Fietta A. M. Ferrari M. Rasini E. Bombelli R. Carcano E. (2007). Human CD4+CD25+ regulatory T cells selectively express tyrosine hydroxylase and contain endogenous catecholamines subserving an autocrine/paracrine inhibitory functional loop. *Blood* 109 632–642. 10.1182/blood-2006-01-028423 16985181

[B38] Costa R. Santa-Maria C. A. Scholtens D. M. Jain S. Flaum L. Gradishar W. J. (2017). A pilot study of cabergoline for the treatment of metastatic breast cancer. *Breast Cancer Res. Treat.* 165 585–592. 10.1007/s10549-017-4370-x 28674764

[B39] Covenas R. Munoz M. (2014). Cancer progression and substance P. *Histol. Histopathol.* 29 881–890. 10.14670/HH-29.881 24535838

[B40] Cunin P. Caillon A. Corvaisier M. Garo E. Scotet M. Blanchard S. (2011). The tachykinins substance P and hemokinin-1 favor the generation of human memory Th17 cells by inducing IL-1beta, IL-23, and TNF-like 1A expression by monocytes. *J. Immunol.* 186 4175–4182. 10.4049/jimmunol.1002535 21368235

[B41] Daher C. Vimeux L. Stoeva R. Peranzoni E. Bismuth G. Wieduwild E. (2019). Blockade of beta-adrenergic receptors improves CD8(+) T-cell Priming and cancer vaccine efficacy. *Cancer Immunol. Res.* 7 1849–1863. 10.1158/2326-6066.CIR-18-0833 31527069

[B42] De Giorgi V. Grazzini M. Benemei S. Marchionni N. Botteri E. Pennacchioli E. (2018). Propranolol for off-label treatment of patients with melanoma: results from a cohort study. *JAMA Oncol.* 4:e172908. 10.1001/jamaoncol.2017.2908 28973254 PMC5838568

[B43] de las Casas-Engel M. Dominguez-Soto A. Sierra-Filardi E. Bragado R. Nieto C. Puig-Kroger A. (2013). Serotonin skews human macrophage polarization through HTR2B and HTR7. *J. Immunol.* 190 2301–2310. 10.4049/jimmunol.1201133 23355731

[B44] Dey P. Li J. Zhang J. Chaurasiya S. Strom A. Wang H. (2020). Oncogenic KRAS-driven metabolic reprogramming in pancreatic cancer cells utilizes cytokines from the tumor microenvironment. *Cancer Discov.* 10 608–625. 10.1158/2159-8290.CD-19-0297 32046984 PMC7125035

[B45] Dimitrov S. Lange T. Gouttefangeas C. Jensen A. T. R. Szczepanski M. Lehnnolz J. (2019). Galphas-coupled receptor signaling and sleep regulate integrin activation of human antigen-specific T cells. *J. Exp. Med.* 216 517–526. 10.1084/jem.20181169 30755455 PMC6400544

[B46] Durk T. Panther E. Muller T. Sorichter S. Ferrari D. Pizzirani C. (2005). 5-Hydroxytryptamine modulates cytokine and chemokine production in LPS-primed human monocytes via stimulation of different 5-HTR subtypes. *Int. Immunol.* 17 599–606. 10.1093/intimm/dxh242 15802305

[B47] Elyada E. Bolisetty M. Laise P. Flynn W. F. Courtois E. T. Burkhart R. A. (2019). Cross-species single-cell analysis of pancreatic ductal adenocarcinoma reveals antigen-presenting cancer-associated fibroblasts. *Cancer Discov.* 9 1102–1123. 10.1158/2159-8290.CD-19-0094 31197017 PMC6727976

[B48] Entschladen F. Palm D. Niggemann B. Zaenker K. S. (2008). The cancer’s nervous tooth: considering the neuronal crosstalk within tumors. *Semin. Cancer Biol.* 18 171–175. 10.1016/j.semcancer.2007.12.004 18249004

[B49] Feske S. Skolnik E. Y. Prakriya M. (2012). Ion channels and transporters in lymphocyte function and immunity. *Nat. Rev. Immunol.* 12 532–547. 10.1038/nri3233 22699833 PMC3670817

[B50] Figueroa C. Galvez-Cancino F. Oyarce C. Contreras F. Prado C. Valeria C. (2017). Inhibition of dopamine receptor D3 signaling in dendritic cells increases antigen cross-presentation to CD8(+) T-cells favoring anti-tumor immunity. *J. Neuroimmunol.* 303 99–107. 10.1016/j.jneuroim.2016.12.014 28077213

[B51] Fox F. E. Kubin M. Cassin M. Niu Z. Hosoi J. Torii H. (1997). Calcitonin gene-related peptide inhibits proliferation and antigen presentation by human peripheral blood mononuclear cells: effects on B7, interleukin 10, and interleukin 12. *J. Invest. Dermatol.* 108 43–48. 10.1111/1523-1747.ep12285627 8980285

[B52] Francis T. Graf A. Hodges K. Kennedy L. Hargrove L. Price M. (2013). Histamine regulation of pancreatitis and pancreatic cancer: a review of recent findings. *Hepatobiliary Surg. Nutr.* 2 216–226. 10.3978/j.issn.2304-3881.2013.08.06 24570946 PMC3924685

[B53] Fu W. X. Qin B. Zhou A. P. Yu Q. Y. Huang Q. J. Liang Z. F. (2011). Regulation of NK92-MI cell cytotoxicity by substance P. *Scand. J. Immunol.* 74 107–113. 10.1111/j.1365-3083.2011.02550.x 21375557

[B54] Gandhi S. Pandey M. R. Attwood K. Ji W. Witkiewicz A. K. Knudsen E. S. (2021). Phase I clinical trial of combination propranolol and pembrolizumab in locally advanced and metastatic melanoma: safety, tolerability, and preliminary evidence of antitumor activity. *Clin. Cancer Res.* 27 87–95. 10.1158/1078-0432.CCR-20-2381 33127652 PMC7785669

[B55] Ghosh M. C. Mondal A. C. Basu S. Banerjee S. Majumder J. Bhattacharya D. (2003). Dopamine inhibits cytokine release and expression of tyrosine kinases, Lck and Fyn in activated T cells. *Int. Immunopharmacol.* 3 1019–1026. 10.1016/S1567-5769(03)00100-0 12810359

[B56] Gobin V. Van Steendam K. Denys D. Deforce D. (2014). Selective serotonin reuptake inhibitors as a novel class of immunosuppressants. *Int. Immunopharmacol.* 20 148–156. 10.1016/j.intimp.2014.02.030 24613205

[B57] Gotzsche C. R. Woldbye D. P. (2016). The role of NPY in learning and memory. *Neuropeptides* 55 79–89. 10.1016/j.npep.2015.09.010 26454711

[B58] Graff R. M. Kunz H. E. Agha N. H. Baker F. L. Laughlin M. Bigley A. B. (2018). beta2-Adrenergic receptor signaling mediates the preferential mobilization of differentiated subsets of CD8+ T-cells, NK-cells and non-classical monocytes in response to acute exercise in humans. *Brain Behav. Immun.* 74 143–153. 10.1016/j.bbi.2018.08.017 30172948 PMC12977291

[B59] Grytli H. H. Fagerland M. W. Fossa S. D. Tasken K. A. (2013a). Reply to Chris R. Cardwell, Samy Suissa and Liam J. Murray’s letter to the editor re: Helene Hartvedt Grytli, Morten Wang Fagerland, Sophie D. Fossa, Kristin Austlid Tasken. Association between use of beta-blockers and prostate cancer-specific survival: a cohort study of 3561 prostate cancer patients with high-risk or metastatic disease. Eur Urol. in press. http://dx.doi.org/10.1016/j.eururo.2013.01.007. *Eur. Urol.* 64 e11–e12. 10.1016/j.eururo.2013.03.045 23582878

[B60] Grytli H. H. Fagerland M. W. Fossa S. D. Tasken K. A. Haheim L. L. (2013b). Use of beta-blockers is associated with prostate cancer-specific survival in prostate cancer patients on androgen deprivation therapy. *Prostate* 73 250–260. 10.1002/pros.22564 22821802

[B61] Guo K. Ma Q. Li J. Wang Z. Shan T. Li W. (2013). Interaction of the sympathetic nerve with pancreatic cancer cells promotes perineural invasion through the activation of STAT3 signaling. *Mol. Cancer Ther.* 12 264–273. 10.1158/1535-7163.MCT-12-0809 23288783

[B62] Gurbuz N. Ashour A. A. Alpay S. N. Ozpolat B. (2014). Down-regulation of 5-HT1B and 5-HT1D receptors inhibits proliferation, clonogenicity and invasion of human pancreatic cancer cells. *PLoS One* 9:e105245. 10.1371/journal.pone.0105245 25170871 PMC4149367

[B63] Haas H. L. Sergeeva O. A. Selbach O. (2008). Histamine in the nervous system. *Physiol. Rev.* 88 1183–1241. 10.1152/physrev.00043.2007 18626069

[B64] Halbrook C. J. Pontious C. Kovalenko I. Lapienyte L. Dreyer S. Lee H. J. (2019). Macrophage-released pyrimidines inhibit gemcitabine therapy in pancreatic cancer. *Cell Metab.* 29 1390–1399.e6. 10.1016/j.cmet.2019.02.001 30827862 PMC6602533

[B65] Han L. Ma J. Duan W. Zhang L. Yu S. Xu Q. (2016). Pancreatic stellate cells contribute pancreatic cancer pain via activation of sHH signaling pathway. *Oncotarget* 7 18146–18158. 10.18632/oncotarget.7776 26934446 PMC4951278

[B66] Hanoun M. Maryanovich M. Arnal-Estape A. Frenette P. S. (2015). Neural regulation of hematopoiesis, inflammation, and cancer. *Neuron* 86 360–373. 10.1016/j.neuron.2015.01.026 25905810 PMC4416657

[B67] Heitz P. Polak J. M. Timson D. M. Pearse A. G. (1976). Enterochromaffin cells as the endocrine source of gastrointestinal substance P. *Histochemistry* 49 343–347. 10.1007/BF00496138 791906

[B68] Herner A. Sauliunaite D. Michalski C. W. Erkan M. De Oliveira T. Abiatari I. (2011). Glutamate increases pancreatic cancer cell invasion and migration via AMPA receptor activation and Kras-MAPK signaling. *Int. J. Cancer* 129 2349–2359. 10.1002/ijc.25898 21207374

[B69] Herr N. Bode C. Duerschmied D. (2017). The effects of serotonin in immune cells. *Front. Cardiovasc. Med.* 4:48. 10.3389/fcvm.2017.00048 28775986 PMC5517399

[B70] Ho W. J. Jaffee E. Zheng L. (2020). The tumour microenvironment in pancreatic cancer–clinical challenges and opportunities. *Nat. Rev. Clin. Oncol.* 17 527–540. 10.1038/s41571-020-0363-5 32398706 PMC7442729

[B71] Hoeppner L. H. Wang Y. Sharma A. Javeed N. Van Keulen V. P. Wang E. (2015). Dopamine D2 receptor agonists inhibit lung cancer progression by reducing angiogenesis and tumor infiltrating myeloid derived suppressor cells. *Mol. Oncol.* 9 270–281. 10.1016/j.molonc.2014.08.008 25226814 PMC4277897

[B72] Holzmann B. (2013). Antiinflammatory activities of CGRP modulating innate immune responses in health and disease. *Curr. Protein Pept. Sci.* 14 268–274. 10.2174/13892037113149990046 23745695

[B73] Hondermarck H. Jobling P. (2018). The sympathetic nervous system drives tumor angiogenesis. *Trends Cancer* 4 93–94. 10.1016/j.trecan.2017.11.008 29458965

[B74] Hosein A. N. Brekken R. A. Maitra A. (2020). Pancreatic cancer stroma: an update on therapeutic targeting strategies. *Nat. Rev. Gastroenterol. Hepatol.* 17 487–505. 10.1038/s41575-020-0300-1 32393771 PMC8284850

[B75] Huang C. Li Y. Guo Y. Zhang Z. Lian G. Chen Y. (2018). MMP1/PAR1/SP/NK1R paracrine loop modulates early perineural invasion of pancreatic cancer cells. *Theranostics* 8 3074–3086. 10.7150/thno.24281 29896303 PMC5996366

[B76] Huang X. Y. Wang H. C. Yuan Z. Huang J. Zheng Q. (2012). Norepinephrine stimulates pancreatic cancer cell proliferation, migration and invasion via beta-adrenergic receptor-dependent activation of P38/MAPK pathway. *Hepatogastroenterology* 59 889–893. 10.5754/hge11476 22020907

[B77] Huang Y. Qiu A. W. Peng Y. P. Liu Y. Huang H. W. Qiu Y. H. (2010). Roles of dopamine receptor subtypes in mediating modulation of T lymphocyte function. *Neuro Endocrinol. Lett.* 31 782–791. 21196914

[B78] Hujber Z. Horvath G. Petovari G. Krencz I. Danko T. Meszaros K. (2018). GABA, glutamine, glutamate oxidation and succinic semialdehyde dehydrogenase expression in human gliomas. *J. Exp. Clin. Cancer Res.* 37:271. 10.1186/s13046-018-0946-5 30404651 PMC6223071

[B79] Idzko M. Panther E. Stratz C. Muller T. Bayer H. Zissel G. (2004). The serotoninergic receptors of human dendritic cells: identification and coupling to cytokine release. *J. Immunol.* 172 6011–6019. 10.4049/jimmunol.172.10.6011 15128784

[B80] Ikeda Y. Takei H. Matsumoto C. Mase A. Yamamoto M. Takeda S. (2007). Administration of substance P during a primary immune response amplifies the secondary immune response via a long-lasting effect on CD8+ T lymphocytes. *Arch. Dermatol. Res.* 299 345–351. 10.1007/s00403-007-0767-4 17643253

[B81] Ilani T. Strous R. D. Fuchs S. (2004). Dopaminergic regulation of immune cells via D3 dopamine receptor: a pathway mediated by activated T cells. *FASEB J.* 18 1600–1602. 10.1096/fj.04-1652fje 15319371

[B82] Inbar S. Neeman E. Avraham R. Benish M. Rosenne E. Ben-Eliyahu S. (2011). Do stress responses promote leukemia progression? An animal study suggesting a role for epinephrine and prostaglandin-E2 through reduced NK activity. *PLoS One* 6:e19246. 10.1371/journal.pone.0019246 21559428 PMC3084788

[B83] Inoue M. Okazaki T. Kitazono T. Mizushima M. Omata M. Ozaki S. (2011). Regulation of antigen-specific CTL and Th1 cell activation through 5-Hydroxytryptamine 2A receptor. *Int. Immunopharmacol.* 11 67–73. 10.1016/j.intimp.2010.10.007 20971187

[B84] Iovino M. Messana T. De Pergola G. Iovino E. Guastamacchia E. Giagulli V. A. (2019). Vigilance states: central neural pathways, neurotransmitters and neurohormones. *Endocr. Metab. Immune Disord. Drug Targets* 19 26–37. 10.2174/1871530318666180816115720 30113008

[B85] Izeboud C. A. Mocking J. A. Monshouwer M. van Miert A. S. Witkamp R. F. (1999). Participation of beta-adrenergic receptors on macrophages in modulation of LPS-induced cytokine release. *J. Recept. Signal Transduct. Res.* 19 191–202. 10.3109/10799899909036645 10071758

[B86] Jandaghi P. Najafabadi H. S. Bauer A. S. Papadakis A. I. Fassan M. Hall A. (2016). Expression of DRD2 is increased in human pancreatic ductal adenocarcinoma and inhibitors slow tumor growth in mice. *Gastroenterology* 151 1218–1231. 10.1053/j.gastro.2016.08.040 27578530

[B87] Janelsins B. M. Sumpter T. L. Tkacheva O. A. Rojas-Canales D. M. Erdos G. Mathers A. R. (2013). Neurokinin-1 receptor agonists bias therapeutic dendritic cells to induce type 1 immunity by licensing host dendritic cells to produce IL-12. *Blood* 121 2923–2933. 10.1182/blood-2012-07-446054 23365459 PMC3624938

[B88] Jiang S. H. Li J. Dong F. Y. Yang J. Y. Liu D. J. Yang X. M. (2017). Increased serotonin signaling contributes to the warburg effect in pancreatic tumor cells under metabolic stress and promotes growth of pancreatic tumors in mice. *Gastroenterology* 153 277–291.e19. 10.1053/j.gastro.2017.03.008 28315323

[B89] Jiang S. H. Zhu L. L. Zhang M. Li R. K. Yang Q. Yan J. Y. (2019). GABRP regulates chemokine signalling, macrophage recruitment and tumour progression in pancreatic cancer through tuning KCNN4-mediated Ca(2+) signalling in a GABA-independent manner. *Gut* 68 1994–2006. 10.1136/gutjnl-2018-317479 30826748

[B90] Jurcak N. Zheng L. (2019). Signaling in the microenvironment of pancreatic cancer: Transmitting along the nerve. *Pharmacol. Ther.* 200 126–134. 10.1016/j.pharmthera.2019.04.010 31047906 PMC6626552

[B91] Katoh N. Soga F. Nara T. Tamagawa-Mineoka R. Nin M. Kotani H. (2006). Effect of serotonin on the differentiation of human monocytes into dendritic cells. *Clin. Exp. Immunol.* 146 354–361. 10.1111/j.1365-2249.2006.03197.x 17034589 PMC1942053

[B92] Kawashima K. Fujii T. (2003). The lymphocytic cholinergic system and its biological function. *Life Sci.* 72 2101–2109. 10.1016/s0024-3205(03)00068-7 12628464

[B93] Kawashima K. Fujii T. (2004). Expression of non-neuronal acetylcholine in lymphocytes and its contribution to the regulation of immune function. *Front. Biosci.* 9:2063–2085. 10.2741/1390 15353271

[B94] Kebabian J. W. (1978). Multiple classes of dopamine receptors in mammalian central nervous system: the involvement of dopamine-sensitive adenylyl cyclase. *Life Sci.* 23 479–483. 10.1016/0024-3205(78)90157-1 357876

[B95] Kim J. I. Ganesan S. Luo S. X. Wu Y. W. Park E. Huang E. J. (2015). Aldehyde dehydrogenase 1a1 mediates a GABA synthesis pathway in midbrain dopaminergic neurons. *Science* 350 102–106. 10.1126/science.aac4690 26430123 PMC4725325

[B96] Kim T. H. Ly C. Christodoulides A. Nowell C. J. Gunning P. W. Sloan E. K. (2019). Stress hormone signaling through beta-adrenergic receptors regulates macrophage mechanotype and function. *FASEB J.* 33 3997–4006. 10.1096/fj.201801429RR 30509116 PMC6404566

[B97] Kim-Fuchs C. Le C. P. Pimentel M. A. Shackleford D. Ferrari D. Angst E. (2014). Chronic stress accelerates pancreatic cancer growth and invasion: a critical role for beta-adrenergic signaling in the pancreatic microenvironment. *Brain Behav. Immun.* 40 40–47. 10.1016/j.bbi.2014.02.019 24650449 PMC4102665

[B98] Kurtulus S. Madi A. Escobar G. Klapholz M. Nyman J. Christian E. (2019). Checkpoint blockade immunotherapy induces dynamic changes in PD-1(−)CD8(+) tumor-infiltrating T cells. *Immunity* 50 181–194.e6. 10.1016/j.immuni.2018.11.014 30635236 PMC6336113

[B99] Kushnir-Sukhov N. M. Gilfillan A. M. Coleman J. W. Brown J. M. Bruening S. Toth M. (2006). 5-hydroxytryptamine induces mast cell adhesion and migration. *J. Immunol.* 177 6422–6432. 10.4049/jimmunol.177.9.6422 17056574

[B100] Lang K. Drell T. L. Niggemann B. Zanker K. S. Entschladen F. (2003). Neurotransmitters regulate the migration and cytotoxicity in natural killer cells. *Immunol. Lett.* 90 165–172. 10.1016/j.imlet.2003.09.004 14687720

[B101] Leone R. D. Horton M. R. Powell J. D. (2015). Something in the air: hyperoxic conditioning of the tumor microenvironment for enhanced immunotherapy. *Cancer Cell* 27 435–436. 10.1016/j.ccell.2015.03.014 25873169 PMC4696011

[B102] Lev A. Lulla A. R. Wagner J. Ralff M. D. Kiehl J. B. Zhou Y. (2017). Anti-pancreatic cancer activity of ONC212 involves the unfolded protein response (UPR) and is reduced by IGF1-R and GRP78/BIP. *Oncotarget* 8 81776–81793. 10.18632/oncotarget.20819 29137221 PMC5669847

[B103] Levite M. (2008). Neurotransmitters activate T-cells and elicit crucial functions via neurotransmitter receptors. *Curr. Opin. Pharmacol.* 8 460–471. 10.1016/j.coph.2008.05.001 18579442

[B104] Levite M. (2016). Dopamine and T cells: dopamine receptors and potent effects on T cells, dopamine production in T cells, and abnormalities in the dopaminergic system in T cells in autoimmune, neurological and psychiatric diseases. *Acta Physiol. (Oxf.)* 216 42–89. 10.1111/apha.12476 25728499

[B105] Li X. Ma G. Ma Q. Li W. Liu J. Han L. (2013). Neurotransmitter substance P mediates pancreatic cancer perineural invasion via NK-1R in cancer cells. *Mol. Cancer Res.* 11 294–302. 10.1158/1541-7786.MCR-12-0609 23345604 PMC3709020

[B106] Li X. Wang Z. Ma Q. Xu Q. Liu H. Duan W. (2014). Sonic hedgehog paracrine signaling activates stromal cells to promote perineural invasion in pancreatic cancer. *Clin. Cancer Res.* 20 4326–4338. 10.1158/1078-0432.CCR-13-3426 24947933

[B107] Lifantseva N. V. Koneeva T. O. Voronezhskaya E. E. Melnikova V. I. (2017). Expression of components of the serotonergic system in the developing rat thymus. *Dokl. Biochem. Biophys.* 477 401–404. 10.1134/S1607672917060151 29297119

[B108] Lifantseva N. V. Koneeva T. O. Voronova S. N. Zakharova L. A. Melnikova V. I. (2016). The inhibition of dopamine synthesis in fetuses changes the pattern of T-lymphocyte maturation in the thymus of adult rats. *Dokl. Biochem. Biophys.* 470 342–344. 10.1134/S1607672916050082 27817026

[B109] Ligorio M. Sil S. Malagon-Lopez J. Nieman L. T. Misale S. Di Pilato M. (2019). Stromal microenvironment shapes the intratumoral architecture of pancreatic cancer. *Cell* 178 160–175.e27. 10.1016/j.cell.2019.05.012 31155233 PMC6697165

[B110] Lin Y. Xu J. Lan H. (2019). Tumor-associated macrophages in tumor metastasis: biological roles and clinical therapeutic applications. *J. Hematol. Oncol.* 12:76. 10.1186/s13045-019-0760-3 31300030 PMC6626377

[B111] Liou G. Y. Bastea L. Fleming A. Doppler H. Edenfield B. H. Dawson D. W. (2017). The presence of interleukin-13 at pancreatic ADM/PanIN lesions alters macrophage populations and mediates pancreatic tumorigenesis. *Cell Rep.* 19 1322–1333. 10.1016/j.celrep.2017.04.052 28514653 PMC5510483

[B112] Liu C. Goel P. Kaeser P. S. (2021). Spatial and temporal scales of dopamine transmission. *Nat. Rev. Neurosci.* 22 345–358. 10.1038/s41583-021-00455-7 33837376 PMC8220193

[B113] Liu Q. Zhang R. Zhang X. Liu J. Wu H. Li Y. (2021). Dopamine improves chemotherapeutic efficacy for pancreatic cancer by regulating macrophage-derived inflammations. *Cancer Immunol. Immunother.* 10.1007/s00262-020-02816-0 [Epub ahead of print]. 33454798 PMC10991349

[B114] Liu Y. Zhang H. Wang Z. Wu P. Gong W. (2019). 5-Hydroxytryptamine1a receptors on tumour cells induce immune evasion in lung adenocarcinoma patients with depression via autophagy/pSTAT3. *Eur. J. Cancer* 114 8–24. 10.1016/j.ejca.2019.03.017 31009821

[B115] Liu Z. Murphy S. F. Huang J. Zhao L. Hall C. C. Schaeffer A. J. (2020). A novel immunocompetent model of metastatic prostate cancer-induced bone pain. *Prostate* 80 782–794. 10.1002/pros.23993 32407603 PMC7375026

[B116] Livingstone E. Hollestein L. M. van Herk-Sukel M. P. van de Poll-Franse L. Nijsten T. Schadendorf D. (2013). beta-Blocker use and all-cause mortality of melanoma patients: results from a population-based Dutch cohort study. *Eur. J. Cancer* 49 3863–3871. 10.1016/j.ejca.2013.07.141 23942335

[B117] Magnon C. Hall S. J. Lin J. Xue X. Gerber L. Freedland S. J. (2013). Autonomic nerve development contributes to prostate cancer progression. *Science* 341:1236361. 10.1126/science.1236361 23846904

[B118] Marino F. Cosentino M. (2013). Adrenergic modulation of immune cells: an update. *Amino Acids* 45 55–71. 10.1007/s00726-011-1186-6 22160285

[B119] Mashaghi A. Marmalidou A. Tehrani M. Grace P. M. Pothoulakis C. Dana R. (2016). Neuropeptide substance P and the immune response. *Cell. Mol. Life Sci.* 73 4249–4264. 10.1007/s00018-016-2293-z 27314883 PMC5056132

[B120] McBurney-Lin J. Lu J. Zuo Y. Yang H. (2019). Locus coeruleus-norepinephrine modulation of sensory processing and perception: a focused review. *Neurosci. Biobehav. Rev.* 105 190–199. 10.1016/j.neubiorev.2019.06.009 31260703 PMC6742544

[B121] Medeiros P. J. Jackson D. N. (2013). Neuropeptide Y Y5-receptor activation on breast cancer cells acts as a paracrine system that stimulates VEGF expression and secretion to promote angiogenesis. *Peptides* 48 106–113. 10.1016/j.peptides.2013.07.029 23932937

[B122] Melamed R. Rosenne E. Shakhar K. Schwartz Y. Abudarham N. Ben-Eliyahu S. (2005). Marginating pulmonary-NK activity and resistance to experimental tumor metastasis: suppression by surgery and the prophylactic use of a beta-adrenergic antagonist and a prostaglandin synthesis inhibitor. *Brain Behav. Immun.* 19 114–126. 10.1016/j.bbi.2004.07.004 15664784

[B123] Meron G. Tishler Y. Shaashua L. Rosenne E. Levi B. Melamed R. (2013). PGE2 suppresses NK activity in vivo directly and through adrenal hormones: effects that cannot be reflected by ex vivo assessment of NK cytotoxicity. *Brain Behav. Immun.* 28 128–138. 10.1016/j.bbi.2012.11.003 23153554 PMC3641317

[B124] Messlinger K. (2018). The big CGRP flood–sources, sinks and signalling sites in the trigeminovascular system. *J. Headache Pain* 19:22. 10.1186/s10194-018-0848-0 29532195 PMC5847494

[B125] Migliarini S. Pacini G. Pelosi B. Lunardi G. Pasqualetti M. (2013). Lack of brain serotonin affects postnatal development and serotonergic neuronal circuitry formation. *Mol. Psychiatry* 18 1106–1118. 10.1038/mp.2012.128 23007167

[B126] Mikulski Z. Zaslona Z. Cakarova L. Hartmann P. Wilhelm J. Tecott L. H. (2010). Serotonin activates murine alveolar macrophages through 5-HT2C receptors. *Am. J. Physiol. Lung Cell. Mol. Physiol.* 299 L272–L280. 10.1152/ajplung.00032.2010 20495077

[B127] Mirlekar B. Michaud D. Lee S. J. Kren N. P. Harris C. Greene K. (2020). B cell-derived IL35 drives STAT3-dependent CD8(+) T-cell exclusion in pancreatic cancer. *Cancer Immunol. Res.* 8 292–308. 10.1158/2326-6066.CIR-19-0349 32024640 PMC7056532

[B128] Misto A. Provensi G. Vozella V. Passani M. B. Piomelli D. (2019). Mast cell-derived histamine regulates liver ketogenesis via oleoylethanolamide signaling. *Cell Metab.* 29 91–102.e5. 10.1016/j.cmet.2018.09.014 30318340

[B129] Mohammadpour H. MacDonald C. R. Qiao G. Chen M. Dong B. Hylander B. L. (2019). beta2 adrenergic receptor-mediated signaling regulates the immunosuppressive potential of myeloid-derived suppressor cells. *J. Clin. Invest.* 129 5537–5552. 10.1172/JCI129502 31566578 PMC6877316

[B130] Monje M. Borniger J. C. D’Silva N. J. Deneen B. Dirks P. B. Fattahi F. (2020). Roadmap for the emerging field of cancer neuroscience. *Cell* 181 219–222. 10.1016/j.cell.2020.03.034 32302564 PMC7286095

[B131] Moon J. H. Kim Y. G. Kim K. Osonoi S. Wang S. Saunders D. C. (2020). Serotonin regulates adult beta-cell mass by stimulating perinatal beta-cell proliferation. *Diabetes* 69 205–214. 10.2337/db19-0546 31806625 PMC6971487

[B132] Munoz M. Covenas R. (2014). Involvement of substance P and the NK-1 receptor in pancreatic cancer. *World J. Gastroenterol.* 20 2321–2334. 10.3748/wjg.v20.i9.2321 24605029 PMC3942835

[B133] Munoz M. Covenas R. (2015). Targeting NK-1 receptors to prevent and treat pancreatic cancer: a new therapeutic approach. *Cancers (Basel)* 7 1215–1232. 10.3390/cancers7030832 26154566 PMC4586765

[B134] Munoz M. Rosso M. Covenas R. (2011). The NK-1 receptor: a new target in cancer therapy. *Curr. Drug Targets* 12 909–921. 10.2174/138945011795528796 21226668

[B135] Muthu K. Iyer S. He L. K. Szilagyi A. Gamelli R. L. Shankar R. (2007). Murine hematopoietic stem cells and progenitors express adrenergic receptors. *J. Neuroimmunol.* 186 27–36. 10.1016/j.jneuroim.2007.02.007 17428548 PMC2020805

[B136] Nakai A. Hayano Y. Furuta F. Noda M. Suzuki K. (2014). Control of lymphocyte egress from lymph nodes through beta2-adrenergic receptors. *J. Exp. Med.* 211 2583–2598. 10.1084/jem.20141132 25422496 PMC4267238

[B137] Nakamura K. Sato T. Ohashi A. Tsurui H. Hasegawa H. (2008). Role of a serotonin precursor in development of gut microvilli. *Am. J. Pathol.* 172 333–344. 10.2353/ajpath.2008.070358 18202184 PMC2312355

[B138] Nakano K. Higashi T. Takagi R. Hashimoto K. Tanaka Y. Matsushita S. (2009). Dopamine released by dendritic cells polarizes Th2 differentiation. *Int. Immunol.* 21 645–654. 10.1093/intimm/dxp033 19332443

[B139] Nichols H. L. Saffeddine M. Theriot B. S. Hegde A. Polley D. El-Mays T. (2012). beta-Arrestin-2 mediates the proinflammatory effects of proteinase-activated receptor-2 in the airway. *Proc. Natl. Acad. Sci. U.S.A.* 109 16660–16665. 10.1073/pnas.1208881109 23012429 PMC3478622

[B140] Nissen M. D. Sloan E. K. Mattarollo S. R. (2018). beta-adrenergic signaling impairs antitumor CD8(+) T-cell responses to B-cell lymphoma immunotherapy. *Cancer Immunol. Res.* 6 98–109. 10.1158/2326-6066.CIR-17-0401 29146881

[B141] O’Connell P. J. Wang X. Leon-Ponte M. Griffiths C. Pingle S. C. Ahern G. P. (2006). A novel form of immune signaling revealed by transmission of the inflammatory mediator serotonin between dendritic cells and T cells. *Blood* 107 1010–1017. 10.1182/blood-2005-07-2903 16223770 PMC1895901

[B142] Ogawa H. Fujii T. Watanabe Y. Kawashima K. (2003). Expression of multiple mRNA species for choline acetyltransferase in human T-lymphocytes. *Life Sci.* 72 2127–2130. 10.1016/s0024-3205(03)00072-9 12628468

[B143] Oh M. S. Guzner A. Wainwright D. A. Mohindra N. A. Chae Y. K. Behdad A. (2021). The impact of beta blockers on survival outcomes in patients with non-small-cell lung cancer treated with immune checkpoint inhibitors. *Clin. Lung Cancer* 22 e57–e62. 10.1016/j.cllc.2020.07.016 32900613 PMC7785632

[B144] Orrego F. (1979). Criteria for the identification of central neurotransmitters, and their application to studies with some nerve tissue preparations in vitro. *Neuroscience* 4 1037–1057. 10.1016/0306-4522(79)90186-6 40157

[B145] Petrilli V. (2017). The multifaceted roles of inflammasome proteins in cancer. *Curr. Opin. Oncol.* 29 35–40. 10.1097/CCO.0000000000000346 27875342

[B146] Prud’homme G. J. Glinka Y. Wang Q. (2015). Immunological GABAergic interactions and therapeutic applications in autoimmune diseases. *Autoimmun. Rev.* 14 1048–1056. 10.1016/j.autrev.2015.07.011 26226414

[B147] Puleo F. Nicolle R. Blum Y. Cros J. Marisa L. Demetter P. (2018). Stratification of pancreatic ductal adenocarcinomas based on tumor and microenvironment features. *Gastroenterology* 155 1999–2013.e3. 10.1053/j.gastro.2018.08.033 30165049

[B148] Qian B. Z. Pollard J. W. (2010). Macrophage diversity enhances tumor progression and metastasis. *Cell* 141 39–51. 10.1016/j.cell.2010.03.014 20371344 PMC4994190

[B149] Qian W. Lv S. Li J. Chen K. Jiang Z. Cheng L. (2018). Norepinephrine enhances cell viability and invasion, and inhibits apoptosis of pancreatic cancer cells in a Notch1dependent manner. *Oncol. Rep.* 40 3015–3023. 10.3892/or.2018.6696 30226612

[B150] Reijmen E. Vannucci L. De Couck M. De Greve J. Gidron Y. (2018). Therapeutic potential of the vagus nerve in cancer. *Immunol. Lett.* 202 38–43. 10.1016/j.imlet.2018.07.006 30077536

[B151] Renz B. W. Takahashi R. Tanaka T. Macchini M. Hayakawa Y. Dantes Z. (2018a). beta2 adrenergic-neurotrophin feedforward loop promotes pancreatic cancer. *Cancer Cell* 34 863–867. 10.1016/j.ccell.2018.10.010 30423300 PMC6261610

[B152] Renz B. W. Tanaka T. Sunagawa M. Takahashi R. Jiang Z. Macchini M. (2018b). Cholinergic signaling via muscarinic receptors directly and indirectly suppresses pancreatic tumorigenesis and cancer stemness. *Cancer Discov.* 8 1458–1473. 10.1158/2159-8290.CD-18-0046 30185628 PMC6214763

[B153] Ribeiro M. P. Custodio J. B. Santos A. E. (2017). Ionotropic glutamate receptor antagonists and cancer therapy: time to think out of the box? *Cancer Chemother. Pharmacol.* 79 219–225. 10.1007/s00280-016-3129-0 27586965

[B154] Riggan L. Shah S. O’Sullivan T. E. (2021). Arrested development: suppression of NK cell function in the tumor microenvironment. *Clin. Transl. Immunol.* 10:e1238. 10.1002/cti2.1238 33456775 PMC7797224

[B155] Roggero E. Besedovsky H. O. del Rey A. (2011). The role of the sympathetic nervous system in the thymus in health and disease. *Neuroimmunomodulation* 18 339–349. 10.1159/000329581 21952686

[B156] Roghanian A. Fraser C. Kleyman M. Chen J. (2016). B cells promote pancreatic tumorigenesis. *Cancer Discov.* 6 230–232. 10.1158/2159-8290.CD-16-0100 26951836

[B157] Roy R. Singh S. M. Shanker A. Sodhi A. (2000). Mechanism of thymocyte apoptosis induced by serum of tumor-bearing host: the molecular events involved and their inhibition by thymosin alpha-1. *Int. J. Immunopharmacol.* 22 309–321. 10.1016/s0192-0561(99)00087-9 10689104

[B158] Saad A. Goldstein J. Margalit O. Shacham-Shmueli E. Lawrence Y. R. Yang Y. X. (2020). Assessing the effects of beta-blockers on pancreatic cancer risk: a nested case-control study. *Pharmacoepidemiol. Drug Saf.* 29 599–604. 10.1002/pds.4993 32196836

[B159] Sacramento P. M. Monteiro C. Dias A. S. O. Kasahara T. M. Ferreira T. B. Hygino J. (2018). Serotonin decreases the production of Th1/Th17 cytokines and elevates the frequency of regulatory CD4(+) T-cell subsets in multiple sclerosis patients. *Eur. J. Immunol.* 48 1376–1388. 10.1002/eji.201847525 29719048

[B160] Sagara A. Nakata K. Yamashita T. Guan W. Zhong P. Matsumoto S. (2021). New high-throughput screening detects compounds that suppress pancreatic stellate cell activation and attenuate pancreatic cancer growth. *Pancreatology.* 10.1016/j.pan.2021.04.002 [Epub ahead of print]. 33965328

[B161] Saha B. Mondal A. C. Majumder J. Basu S. Dasgupta P. S. (2001). Physiological concentrations of dopamine inhibit the proliferation and cytotoxicity of human CD4+ and CD8+ T cells in vitro: a receptor-mediated mechanism. *Neuroimmunomodulation* 9 23–33. 10.1159/000049004 11435749

[B162] Saloman J. L. Albers K. M. Li D. Hartman D. J. Crawford H. C. Muha E. A. (2016). Ablation of sensory neurons in a genetic model of pancreatic ductal adenocarcinoma slows initiation and progression of cancer. *Proc. Natl. Acad. Sci. U.S.A.* 113 3078–3083. 10.1073/pnas.1512603113 26929329 PMC4801275

[B163] Saponara E. Visentin M. Baschieri F. Seleznik G. Martinelli P. Esposito I. (2018). Serotonin uptake is required for Rac1 activation in Kras-induced acinar-to-ductal metaplasia in the pancreas. *J. Pathol.* 246 352–365. 10.1002/path.5147 30058725

[B164] Sarkar C. Chakroborty D. Basu S. (2013). Neurotransmitters as regulators of tumor angiogenesis and immunity: the role of catecholamines. *J. Neuroimmune Pharmacol.* 8 7–14. 10.1007/s11481-012-9395-7 22886869 PMC3869381

[B165] Sarkar C. Das S. Chakroborty D. Chowdhury U. R. Basu B. Dasgupta P. S. (2006). Cutting edge: stimulation of dopamine D4 receptors induce T cell quiescence by up-regulating Kruppel-like factor-2 expression through inhibition of ERK1/ERK2 phosphorylation. *J. Immunol.* 177 7525–7529. 10.4049/jimmunol.177.11.7525 17114421

[B166] Schuller H. M. Al-Wadei H. A. Ullah M. F. Plummer H. K. III (2012). Regulation of pancreatic cancer by neuropsychological stress responses: a novel target for intervention. *Carcinogenesis* 33 191–196. 10.1093/carcin/bgr251 22072614 PMC3276326

[B167] Shahzad M. M. K. Arevalo J. M. Armaiz-Pena G. N. Lu C. Stone R. L. Moreno-Smith M. (2018). Stress effects on FosB and interleukin-8 (IL8)-driven ovarian cancer growth and metastasis. *J. Biol. Chem.* 293:10041. 10.1074/jbc.AAC118.004299 29959278 PMC6028963

[B168] Sinha S. Fu Y. Y. Grimont A. Ketcham M. Lafaro K. Saglimbeni J. A. (2017). PanIN neuroendocrine cells promote tumorigenesis via neuronal cross-talk. *Cancer Res.* 77 1868–1879. 10.1158/0008-5472.CAN-16-0899-T 28386018 PMC5471615

[B169] Soga F. Katoh N. Inoue T. Kishimoto S. (2007). Serotonin activates human monocytes and prevents apoptosis. *J. Invest. Dermatol.* 127 1947–1955. 10.1038/sj.jid.5700824 17429435

[B170] Soltani N. Qiu H. Aleksic M. Glinka Y. Zhao F. Liu R. (2011). GABA exerts protective and regenerative effects on islet beta cells and reverses diabetes. *Proc. Natl. Acad. Sci. U.S.A.* 108 11692–11697. 10.1073/pnas.1102715108 21709230 PMC3136292

[B171] Song Y. Gan Y. Wang Q. Meng Z. Li G. Shen Y. (2017). Enriching the housing environment for mice enhances their NK cell antitumor immunity via sympathetic nerve-dependent regulation of NKG2D and CCR5. *Cancer Res.* 77 1611–1622. 10.1158/0008-5472.CAN-16-2143 28082402

[B172] Spitzer N. C. (2015). Neurotransmitter switching? No surprise. *Neuron* 86 1131–1144. 10.1016/j.neuron.2015.05.028 26050033 PMC4458710

[B173] Spohn S. N. Mawe G. M. (2017). Non-conventional features of peripheral serotonin signalling–the gut and beyond. *Nat. Rev. Gastroenterol. Hepatol.* 14 412–420. 10.1038/nrgastro.2017.51 28487547 PMC5672796

[B174] Stein M. N. Malhotra J. Tarapore R. S. Malhotra U. Silk A. W. Chan N. (2019). Safety and enhanced immunostimulatory activity of the DRD2 antagonist ONC201 in advanced solid tumor patients with weekly oral administration. *J. Immunother. Cancer* 7:136. 10.1186/s40425-019-0599-8 31118108 PMC6532211

[B175] Sung H. Y. Yang S. D. Ju W. Ahn J. H. (2017). Aberrant epigenetic regulation of GABRP associates with aggressive phenotype of ovarian cancer. *Exp. Mol. Med.* 49:e335. 10.1038/emm.2017.62 28524180 PMC5454450

[B176] Suvas S. (2017). Role of substance p neuropeptide in inflammation, wound healing, and tissue homeostasis. *J. Immunol.* 199 1543–1552. 10.4049/jimmunol.1601751 28827386 PMC5657331

[B177] Takehara A. Hosokawa M. Eguchi H. Ohigashi H. Ishikawa O. Nakamura Y. (2007). Gamma-aminobutyric acid (GABA) stimulates pancreatic cancer growth through overexpressing GABAA receptor pi subunit. *Cancer Res.* 67 9704–9712. 10.1158/0008-5472.CAN-07-2099 17942900

[B178] Talhada D. Rabenstein M. Ruscher K. (2018). The role of dopaminergic immune cell signalling in poststroke inflammation. *Ther. Adv. Neurol. Disord.* 11:1756286418774225. 10.1177/1756286418774225 29774058 PMC5952273

[B179] Tan X. Sivakumar S. Bednarsch J. Wiltberger G. Kather J. N. Niehues J. (2021). Nerve fibers in the tumor microenvironment in neurotropic cancer-pancreatic cancer and cholangiocarcinoma. *Oncogene* 40 899–908. 10.1038/s41388-020-01578-4 33288884 PMC7862068

[B180] Tarr A. J. Powell N. D. Reader B. F. Bhave N. S. Roloson A. L. Carson W. E. III (2012). beta-adrenergic receptor mediated increases in activation and function of natural killer cells following repeated social disruption. *Brain Behav. Immun.* 26 1226–1238. 10.1016/j.bbi.2012.07.002 22796551 PMC3468689

[B181] Thaker P. H. Han L. Y. Kamat A. A. Arevalo J. M. Takahashi R. Lu C. (2006). Chronic stress promotes tumor growth and angiogenesis in a mouse model of ovarian carcinoma. *Nat. Med.* 12 939–944. 10.1038/nm1447 16862152

[B182] Uyttenhove C. Pilotte L. Theate I. Stroobant V. Colau D. Parmentier N. (2003). Evidence for a tumoral immune resistance mechanism based on tryptophan degradation by indoleamine 2,3-dioxygenase. *Nat. Med.* 9 1269–1274. 10.1038/nm934 14502282

[B183] Van Overmeire E. Stijlemans B. Heymann F. Keirsse J. Morias Y. Elkrim Y. (2016). M-CSF and GM-CSF receptor signaling differentially regulate monocyte maturation and macrophage polarization in the tumor microenvironment. *Cancer Res.* 76 35–42. 10.1158/0008-5472.CAN-15-0869 26573801

[B184] Waldmann J. Fendrich V. Reichert M. Hecker A. Bartsch D. K. Padberg W. (2018). Expression of neuropeptide Y and its receptors Y1 and Y2 in pancreatic intraepithelial neoplasia and invasive pancreatic cancer in a transgenic mouse model and human samples of pancreatic cancer. *J. Surg. Res.* 223 230–236. 10.1016/j.jss.2017.11.010 29433879

[B185] Wang T. Huang W. Chen F. (2008). Baclofen, a GABAB receptor agonist, inhibits human hepatocellular carcinoma cell growth in vitro and in vivo. *Life Sci.* 82 536–541. 10.1016/j.lfs.2007.12.014 18222491

[B186] Wang X. Li B. Kim Y. J. Wang Y. C. Li Z. Yu J. (2021). Targeting monoamine oxidase A for T cell-based cancer immunotherapy. *Sci. Immunol.* 6:eabh2383. 10.1126/sciimmunol.abh2383 33990379

[B187] Watanabe Y. Nakayama T. Nagakubo D. Hieshima K. Jin Z. Katou F. (2006). Dopamine selectively induces migration and homing of naive CD8+ T cells via dopamine receptor D3. *J. Immunol.* 176 848–856. 10.4049/jimmunol.176.2.848 16393968

[B188] Wculek S. K. Cueto F. J. Mujal A. M. Melero I. Krummel M. F. Sancho D. (2020). Dendritic cells in cancer immunology and immunotherapy. *Nat. Rev. Immunol.* 20 7–24. 10.1038/s41577-019-0210-z 31467405

[B189] Wheway J. Herzog H. Mackay F. (2007a). NPY and receptors in immune and inflammatory diseases. *Curr. Top. Med. Chem.* 7 1743–1752. 10.2174/156802607782341046 17979783

[B190] Wheway J. Herzog H. Mackay F. (2007b). The Y1 receptor for NPY: a key modulator of the adaptive immune system. *Peptides* 28 453–458. 10.1016/j.peptides.2006.09.030 17240480

[B191] Winkler J. Abisoye-Ogunniyan A. Metcalf K. J. Werb Z. (2020). Concepts of extracellular matrix remodelling in tumour progression and metastasis. *Nat. Commun.* 11:5120. 10.1038/s41467-020-18794-x 33037194 PMC7547708

[B192] Wouters M. C. A. Nelson B. H. (2018). Prognostic Significance of tumor-infiltrating B cells and plasma cells in human cancer. *Clin. Cancer Res.* 24 6125–6135. 10.1158/1078-0432.CCR-18-1481 30049748

[B193] Wu C. Qin X. Du H. Li N. Ren W. Peng Y. (2017). The immunological function of GABAergic system. *Front. Biosci. (Landmark Ed.)* 22 1162–1172. 28199198 10.2741/4539

[B194] Wu H. H. Hwang-Verslues W. W. Lee W. H. Huang C. K. Wei P. C. Chen C. L. (2015). Targeting IL-17B-IL-17RB signaling with an anti-IL-17RB antibody blocks pancreatic cancer metastasis by silencing multiple chemokines. *J. Exp. Med.* 212 333–349. 10.1084/jem.20141702 25732306 PMC4354366

[B195] Wu J. Zhang R. Tang N. Gong Z. Zhou J. Chen Y. (2015). Dopamine inhibits the function of Gr-1+CD115+ myeloid-derived suppressor cells through D1-like receptors and enhances anti-tumor immunity. *J. Leukoc. Biol.* 97 191–200. 10.1189/jlb.5A1113-626RR 25341727 PMC4377827

[B196] Xie L. Moroi Y. Tsuji G. Liu M. Hayashida S. Takahara M. (2010). CD10-bearing fibroblast inhibits matrigel invasive potency of interleukin-1alpha-producing squamous cell carcinoma by diminishing substance P levels in the tumor microenvironment. *Cancer Sci.* 101 2570–2578. 10.1111/j.1349-7006.2010.01735.x 20874839 PMC11158981

[B197] Yan C. Richmond A. (2020). Th9 and Th17 cells: the controversial twins in cancer immunity. *J. Clin. Invest.* 130 3409–3411. 10.1172/JCI138418 32484457 PMC7324204

[B198] Yan Y. Jiang W. Liu L. Wang X. Ding C. Tian Z. (2015). Dopamine controls systemic inflammation through inhibition of NLRP3 inflammasome. *Cell* 160 62–73. 10.1016/j.cell.2014.11.047 25594175

[B199] Yang M. W. Tao L. Y. Jiang Y. S. Yang J. Y. Huo Y. M. Liu D. J. (2020). Perineural invasion reprograms the immune microenvironment through cholinergic signaling in pancreatic ductal adenocarcinoma. *Cancer Res.* 80 1991–2003. 10.1158/0008-5472.CAN-19-2689 32098780

[B200] Yang Q. Yan C. Yin C. Gong Z. (2017). Serotonin activated hepatic stellate cells contribute to sex disparity in hepatocellular carcinoma. *Cell. Mol. Gastroenterol. Hepatol.* 3 484–499. 10.1016/j.jcmgh.2017.01.002 28462385 PMC5403976

[B201] Yin J. Albert R. H. Tretiakova A. P. Jameson B. A. (2006). 5-HT(1B) receptors play a prominent role in the proliferation of T-lymphocytes. *J. Neuroimmunol.* 181 68–81. 10.1016/j.jneuroim.2006.08.004 17011639

[B202] Yu L. J. Wall B. A. Wangari-Talbot J. Chen S. (2017). Metabotropic glutamate receptors in cancer. *Neuropharmacology* 115 193–202. 10.1016/j.neuropharm.2016.02.011 26896755 PMC4987272

[B203] Zahalka A. H. Arnal-Estape A. Maryanovich M. Nakahara F. Cruz C. D. Finley L. W. S. (2017). Adrenergic nerves activate an angio-metabolic switch in prostate cancer. *Science* 358 321–326. 10.1126/science.aah5072 29051371 PMC5783182

[B204] Zahalka A. H. Frenette P. S. (2020). Nerves in cancer. *Nat. Rev. Cancer* 20 143–157. 10.1038/s41568-019-0237-2 31974491 PMC7709871

[B205] Zaidi Z. F. Matthews M. R. (2013). Source and origin of nerve fibres immunoreactive for substance P and calcitonin gene-related peptide in the normal and chronically denervated superior cervical sympathetic ganglion of the rat. *Auton. Neurosci.* 173 28–38. 10.1016/j.autneu.2012.11.002 23167990

[B206] Zhang D. Li X. Yao Z. Wei C. Ning N. Li J. (2014). GABAergic signaling facilitates breast cancer metastasis by promoting ERK1/2-dependent phosphorylation. *Cancer Lett.* 348 100–108. 10.1016/j.canlet.2014.03.006 24657659

[B207] Zhang Y. Lazarus J. Steele N. G. Yan W. Lee H. J. Nwosu Z. C. (2020). Regulatory T-cell Depletion alters the tumor microenvironment and accelerates pancreatic carcinogenesis. *Cancer Discov.* 10 422–439. 10.1158/2159-8290.CD-19-0958 31911451 PMC7224338

[B208] Zhang Y. Velez-Delgado A. Mathew E. Li D. Mendez F. M. Flannagan K. (2017). Myeloid cells are required for PD-1/PD-L1 checkpoint activation and the establishment of an immunosuppressive environment in pancreatic cancer. *Gut* 66 124–136. 10.1136/gutjnl-2016-312078 27402485 PMC5256390

[B209] Zhao C. M. Hayakawa Y. Kodama Y. Muthupalani S. Westphalen C. B. Andersen G. T. (2014). Denervation suppresses gastric tumorigenesis. *Sci. Transl. Med.* 6:250ra115. 10.1126/scitranslmed.3009569 25143365 PMC4374618

[B210] Zhou Z. (1993). [A study of CGRP receptor and its effect on the growth of human pancreatic carcinoma cells]. *Zhongguo Yi Xue Ke Xue Yuan Xue Bao* 15 427–432. 8082251

[B211] Zhu Y. Herndon J. M. Sojka D. K. Kim K. W. Knolhoff B. L. Zuo C. (2017). Tissue-resident macrophages in pancreatic ductal adenocarcinoma originate from embryonic hematopoiesis and promote tumor progression. *Immunity* 47 323–338.e6. 10.1016/j.immuni.2017.07.014 28813661 PMC5578409

